# Pathophysiological Mechanisms in Non-Alcoholic Fatty Liver Disease: From Drivers to Targets

**DOI:** 10.3390/biomedicines10010046

**Published:** 2021-12-26

**Authors:** Alvaro Santos-Laso, María Gutiérrez-Larrañaga, Marta Alonso-Peña, Juan M. Medina, Paula Iruzubieta, María Teresa Arias-Loste, Marcos López-Hoyos, Javier Crespo

**Affiliations:** 1 Department of Gastroenterology and Hepatology, Marqués de Valdecilla University Hospital, Valdecilla Biomedical Research Institute (IDIVAL), 39008 Santander, Spain; malonso@idival.org (M.A.-P.); jmedina@idival.org (J.M.M.); p.iruzubieta@gmail.com (P.I.); ariasloste@gmail.com (M.T.A.-L.); 2 Department of Immunology, Marqués de Valdecilla University Hospital, Valdecilla Biomedical Research Institute (IDIVAL), 39008 Santander, Spain; maria.gutierrezl@scsalud.es (M.G.-L.); marcos.lopez@scsalud.es (M.L.-H.); 3 National Institute for the Study of Liver and Gastrointestinal Diseases (CIBERehd, Instituto de Salud Carlos III), 28029 Madrid, Spain

**Keywords:** non-alcoholic fatty liver disease, pathogenesis, metabolism, inflammation, dysbiosis, pharmacological targets

## Abstract

Non-alcoholic fatty liver disease (NAFLD) is characterized by the excessive and detrimental accumulation of liver fat as a result of high-caloric intake and/or cellular and molecular abnormalities. The prevalence of this pathological event is increasing worldwide, and is intimately associated with obesity and type 2 diabetes mellitus, among other comorbidities. To date, only therapeutic strategies based on lifestyle changes have exhibited a beneficial impact on patients with NAFLD, but unfortunately this approach is often difficult to implement, and shows poor long-term adherence. For this reason, great efforts are being made to elucidate and integrate the underlying pathological molecular mechanism, and to identify novel and promising druggable targets for therapy. In this regard, a large number of clinical trials testing different potential compounds have been performed, albeit with no conclusive results yet. Importantly, many other clinical trials are currently underway with results expected in the near future. Here, we summarize the key aspects of NAFLD pathogenesis and therapeutic targets in this frequent disorder, highlighting the most recent advances in the field and future research directions.

## 1. Introduction

Non-alcoholic fatty liver disease (NAFLD) is the most common chronic liver disease in Western countries, with an estimated global prevalence of 25%. It includes a wide spectrum of liver injuries whose distinctive feature is the accumulation of intrahepatic fat, especially triglycerides (TGs) [1]. Particularly, the spectrum of NAFLD ranges from simple steatosis to non-alcoholic steatohepatitis (NASH), which is characterized by a variable grade of inflammation and hepatocellular damage [2,3] and may further progress to more severe hepatic disorders, such as fibrosis, cirrhosis, and hepatocellular carcinoma (HCC) [4]. In addition to liver-related disease, NAFLD is also strongly associated with several extra-hepatic metabolic comorbidities (i.e., metabolic syndrome [MetS], type 2 diabetes mellitus [T2DM], obesity, and hypertension, among others), cardiovascular disease, and chronic kidney disease, raising the mortality rate [5,6]. Recently, an international expert panel has published a consensus statement proposing the renaming of NAFLD to metabolic-associated fatty liver disease (MAFLD) [7]. This is not only a change of name, but also a set of new “positive” criteria for the diagnosis of MAFLD, regardless of alcohol consumption or other concomitant liver diseases, given the dramatic rise in the global prevalence of this liver disease. Certainly, this liver disorder is no longer a “histological disease”, abandoning the dichotomous stratification into NAFLD and NASH, which may not capture the full spectrum of the disease course in response to changes in the underlying metabolic dysfunction or to pharmacological interventions [7]. The new definition clearly establishes this disease as a metabolic disorder; however, besides metabolic dysfunction, many other diseases result in hepatic steatosis, such as alcohol- and drug-induced liver injury, and chronic inflammatory diseases. Some real-life implications of this new terminology in clinical practice have been evaluated in the last year [8,9], although its use is not yet widely extended.

Hepatic steatosis may result from dysfunction of multiple pathways regulating lipid entry, synthesis, oxidation, and excretion. Therefore, there are many factors influencing NAFLD initiation and progression, such as environmental exposure, lifestyle, genetic susceptibility, metabolic status, and the microbiome [10]. Accumulation of lipids, especially free fatty acids (FFAs), causes detrimental effects in the hepatocytes, including induction of endoplasmic reticulum stress (ER stress) and the unfolded protein response, oxidative stress, and the subsequent expression of pro-inflammatory cytokines [11,12]. Besides, fatty acids (FAs) can induce hepatic insulin resistance (IR) [13], and directly activate inflammatory signaling acting as ligands for innate immunity receptors, such as toll-like receptors (TLRs) [14]. Liver-related innate immune responses to excess fat can directly result in hepatocyte apoptosis [15]. However, inflammation may precede steatosis, as inflammatory events may lead to lipid accumulation [16,17]. Therefore, inflammatory processes may play key roles in the pathogenesis of fatty liver diseases. Moreover, treatment strategies might need anti-inflammatory approaches to target this disease successfully. This complexity contributes to the fact that there are currently no approved drug treatments for this pathology [18]. In addition, clinical trials are not controlled for individual genetic predisposition, signal transduction, or metabolic profiles. Patient recruitment for clinical trials is currently based on liver histological involvement, but many pathological pathways can lead to the same histological phenotype. Therefore, clinical trials reporting for NAFLD are suboptimal, limiting our understanding. All these data suggest the existence of different phenotypes within NAFLD that differ in which molecular pathways are predominantly altered, that is inflammatory or metabolic pathways or both, and they probably have a different natural history and liver disease course.

This review is aimed at summarizing current knowledge about the pathophysiological mechanisms involved in the establishment and progression of NAFLD and their effects in the liver, the efforts in developing effective drug treatments attending to these mechanisms, and at discussing the potential stratification of patients to identify the better candidates for each treatment.

## 2. Molecular Mechanisms of Pathogenesis

Lipid accumulation within the liver is the first and better known “hit” responsible for NAFLD initiation and progression [19]. Moreover, an excess of carbohydrates can be converted into FFAs and TGs [3]. Of note, insulin acts as a pivotal regulator of this metabolic step by controlling blood glucose clearance [20]. Contrarily, fructose is metabolized regardless of the levels of this hormone, although it could also induce hyperinsulinemia over time [21]. Therefore, a link between high-sugar diets and insulin resistance (IR) with steatosis is established. In addition, when lipotoxic metabolites accumulate inside the cells, they can cause mitochondria dysfunction by uncoupling oxidative phosphorylation, leading to reactive oxygen species (ROS) production, and potentially generating oxidative stress [11]. In this sense, endoplasmic reticulum stress (ER stress) has also shown to participate in oxidative stress generation in NAFLD [12]. ROS, at the same time, have shown to activate pro-inflammatory pathways, such as NF-kB, either directly or indirectly by ROS-damaged DNA [22].

Around 75% of the hepatic blood supply comes directly from the gut through the portal vein. This blood brings toxins, as well as antigens from potentially harmful external pathogens. Thus, in addition to its role in metabolism and detoxification, the liver is a key immunological organ, acting as a barrier between the external and internal environment [23]. Noteworthy, the portal vein also brings antigens from commensal microbiota and foreign, but harmless, molecules, which have to be tolerated by the immune system. This means that the liver’s immune status is anti-inflammatory or immunotolerant by default, but, at the same time, is able to mount a rapid and robust immune response under the appropriate conditions [23]. Some foreign molecules that reach the liver are microbial-derived metabolites with a characterized impact in the integrity of this organ at different digestive pathologies [24].

Below, we address the main pathogenic mechanisms described to be involved in NAFLD development and progression.

### 2.1. Lipid Metabolism

**Figure 1:**
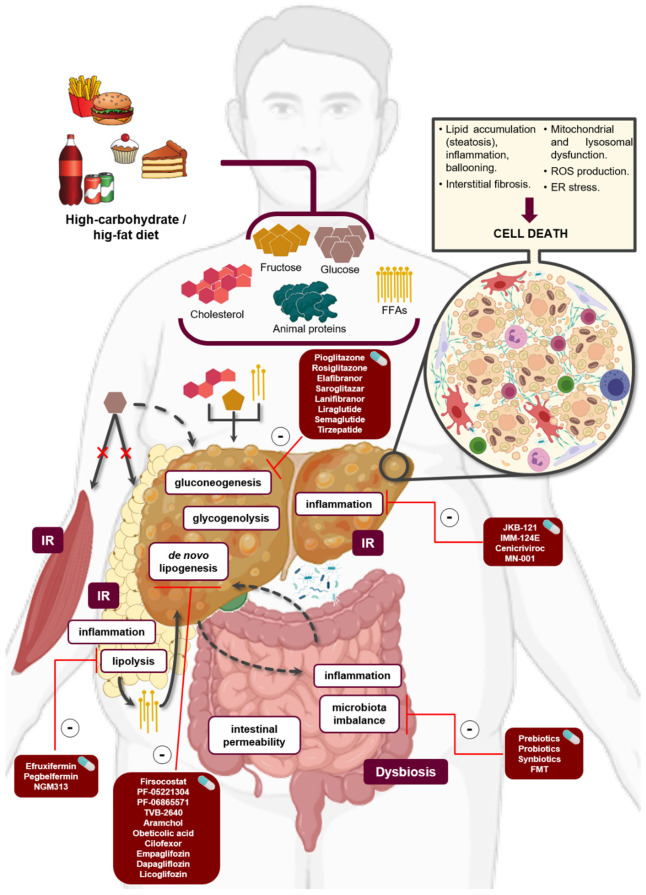
Molecular mechanisms and therapeutic targets involved in NAFLD. High-carbohydrate and/or high-fat diet leads to multisystem dysregulation that exacerbates the gluconeogenesis, glycogenolysis, and *de novo* lipogenesis pathways in the liver, contributing to liver fat accumulation and inflammation. Moreover, dysregulation of the gut-liver-axis emerges as another pathological mechanism in NAFLD. Numerous pharmacological treatments have been developed to act on different processes involved in the onset and progression of the disease. Abbreviations: ER stress, endoplasmic reticulum stress; FFAs, free fatty acids; IR, insulin resistance; ROS, reactive oxygen species. Created with BioRender.com (last access: 22 November 2021).

Despite steatosis being traditionally considered a relatively benign condition, the effects of which are reversible when interventions are performed, hepatic lipotoxicity occurs when the liver capacity to use, store, and export lipids and lipid derivatives is overwhelmed by a massive flux of such molecules from the adipose tissue, gut absorption, or by increased hepatic de novo lipogenesis (DNL). Indeed, 60% of the hepatic FFAs results from adipose tissue TGs after lipolysis, 25% from hepatic DNL, and 15% from dietary FFAs [25] (Figure 1). Certainly, FFAs levels correlate with disease severity [26].

FFAs and TGs could be subsequently biotransformed in hepatotoxic lipids, such as lysophosphatidylcholine (LPC), ceramides, free cholesterol (FC), and bile acids (BAs) [3]. Through transcriptomic analysis, NAFLD and, especially, NASH are characterized by the overexpression of genes associated with lipid metabolism, whereas genes modulating fatty acid (FA) metabolism are downregulated [25]. As mentioned before, mechanisms involved in lipotoxicity include ER stress, c-Jun NH2-terminal kinase (JNK)-induced toxicity, and mitochondrial and lysosomal dysfunction, although the field is rapidly evolving, and most studies are performed in murine models, whereas human studies are limited. For a detailed description, consider reading Mota et al. [3] (Figure 1).

#### 2.1.1. Fatty Acids

Lipidomic studies have described specific changes in the hepatic lipidome in patients with NAFLD. Among FFAs, the hepatic concentrations of saturated fatty acids (SFAs) increase, whereas ω-3 polyunsaturated fatty acids (PUFAs) decrease [25]. SFAs accumulation is positively associated with liver disease severity. In hepatocytes, SFAs stimulate pro-inflammatory cytokine secretion, enhance ER stress, increase ROS, decrease mitochondrial and peroxisome β-oxidation, and induce apoptosis. SFAs stimulate the production and secretion of pro-inflammatory and pro-fibrotic cytokines from Kupffer cells (KCs), and induce pro-inflammatory M1 polarization of macrophages, as described later. Additionally, they stimulate the secretion of chemokines from hepatic stellate cells (HSCs) [25]. In NAFLD, a major dysregulation in the hepatic long-chain FA desaturation processes is observed, resulting in an elevated ω-6 to ω-3 ratio, and increased flux in the ω-6 pathway. ω-6 PUFAs lead to the synthesis of eicosanoids with pro-inflammatory properties, such as prostanglandines, thromboxanes, and leukotrienes. Moreover, high ω-6 to ω-3 ratio can affect cell membrane phospholipid composition, resulting in cell necrosis and extracellular deposition of lipotoxic lipids [25]. On the other hand, TGs generate more hepatocyte steatosis, but FFAs are responsible for higher rates of apoptosis. It appears that TGs represent a defense system against the pro-apoptotic effects of large loads of FFAs in cells [3]. Besides, monounsaturated fatty acids (MUFAs) are lipotoxic, but in a lesser degree compared to SFAs. Thus, a higher ratio of MUFA/SFA may be beneficial due to the lower ability of MUFAs to stimulate ER stress and apoptosis [25].

#### 2.1.2. Compound Lipids

Sphingolipids, glycerophospholipids, and eicosanoids, which exert hepatotoxic capacity, also increase in NAFLD [25]. Ceramides are elevated in NAFLD, and positively correlate with disease severity [25]. An increased circulating concentration of ceramides was seen in peripheral blood samples of obese patients with NASH [3]. Ceramide promotes IR; impairs β-oxidation; induces ROS production, ER stress, and pro-inflammatory cytokine secretion; enhances cholesterol synthesis; induces apoptosis; and stimulates fibrogenesis and angiogenesis [25]. LPC stimulates ER stress, causes mitochondrial dysfunction, and increases apoptosis [3]. The increased transformation of LPC from phosphatidylcholine (PC) leads to rapid depletion of PC, which affects hepatocyte membrane integrity; a high release of lipotoxic lipids; and increased inflammation. Additionally, PC deficiency reduces very low-density lipoprotein (VLDL) secretion, resulting in higher intrahepatic lipid degradation, and the formation of toxic intermediates. Finally, LPC is metabolized by the enzyme autotaxin to phospholipid lysophosphatidic acid, which stimulates liver fibrosis and the development of HCC [25]. LPC appears to be a key instigator of lipotoxicity, and it has been suggested that FFAs could exert cytotoxicity through the generation of LPC [3].

#### 2.1.3. Cholesterol

Finally, hepatic FC accumulates as a result of enhanced synthesis, increased cholesterol de-esterification, and decreased cholesterol export and BA synthesis. It is worth mentioning that diets including higher amounts of FC result in enhanced liver injury compared to high fat diets with poor FC content in preclinical models of NAFLD [27]. Abundant intracellular FC stimulates KCs and HSCs [3]. BAs are the principal route for cholesterol catabolism [28]. Moreover, BAs prevent the overgrowth of bacteria in the gut, and exert a strong antimicrobial role in maintaining gut homeostasis. In addition, it is recognized that circulating BAs can coordinate a wide number of pathways, mediated by specific nuclear receptors. Thus, BAs can regulate lipid and glucose metabolism upon binding to farnesoid X receptor (FXR), which could induce the expression of the small heterodimer partner, promoting the inhibition of sterol regulatory element-binding protein 1c (SREBP-1c), and thus, reducing the hepatic synthesis of TGs. In addition, FXR can limit lipid accumulation in the liver by promoting FA oxidation after the activation of peroxisome proliferator-activated receptor (PPAR) α, and by the induction of plasma VLDL triglyceride clearance [28]. It is important to mention that most studies regarding NAFLD pathophysiological mechanisms are based on animal models, and translation to human physiology should be cautious.

#### 2.1.4. Evidence from Patients

As previously mentioned, the inability of the liver to handle high FFAs is the first trigger of the disease, and it is a direct consequence of obesity. Moreover, different fat distribution patterns in the abdominal area among individuals with a similar BMI may result in different metabolic consequences. Researchers have proven the existence of metabolically benign obese phenotypes that have lower IR and atherosclerosis than their obese counterparts, and metabolically unhealthy obese phenotypes among non-obese individuals [29]. Despite that, some studies showed that the contribution of visceral adipose tissue (VAT) to NAFLD is more significant than waist circumference or total body fat. Subcutaneous fat serves as storage of metabolically benign fat. However, VAT that only makes up for 7–15% of total body fat is associated with low high-density lipoprotein (HDL) levels, hypertriglyceridemia, and IR. A higher VAT-to-subcutaneous fat ratio is associated with increased risk of NAFLD development and advanced fibrosis risk [28]. Besides, increasing VAT volume is a strong independent predictor of hepatic fat accumulation, and is associated with radiographic and serum markers of NAFLD severity [30]. Interestingly, a recent study analyzed the body composition of 138 obese patients by bioelectrical impedance analysis (BIA). Among them, more than half of the study cohort (i.e., 64%) was classified as NASH or borderline NASH according to liver biopsy, detecting an association between higher fat mass, BIA, and histology, and supporting that body fat might be an important parameter for the diagnosis of NASH in obese patients [31].

Although the contribution of lipid metabolism and accumulation in the development of NAFLD in obese patients is clear, the existence and clinical course of the entity known as “lean NAFLD” has been the subject of intense debate. To many, lean NAFLD refers to individuals manifesting the disease in the context of a normal BMI, but having excess VAT and IR, as well as metabolic dysfunction [28]. By this interpretation, lean NAFLD is similar to NAFLD associated with obesity, with IR at its core (See Section 2.2). However, lipid metabolism can play a distinct and important role in these patients. In this regard, patients with lean NAFLD had higher secondary BA levels compared with those with non-lean NAFLD, and present different microbiota changes [28]. As mentioned before, BAs have a pivotal role in hepatic metabolism and the gut-liver axis (GLA). Thus, authors suggest that patients who are lean can adapt metabolically and excrete greater amounts of BAs, whereas their obese counterparts are those less able to excrete adequate amounts of BAs to rid themselves of excess cholesterol. These changes were more profound in those patients with the early stages of liver fibrosis, whereas at later stages, these homeostatic responses might no longer be able to limit inflammation and fibrosis, leading ultimately to long-term adverse outcomes [28]. This line of evidence supports the treatment in earlier stages of the disease.

On the other hand, several attempts have been made to identify genetic risk factors for NAFLD development. More consistent and better-known susceptibility *loci* affect *patatin-like phospholipase domain containing 3* (*PNPLA3)* and *transmembrane 6 superfamily member 2* (*TM6SF2)* [25]. *PNPLA3* encodes adiponutrin, a protein with lipase and acyltransferase activity, expressed in liver and adipose tissue. PNPLA3 variant p.I148M (rs738409) affects hepatic lipid composition by decreasing PUFA transfer from diacylglycerols (DAGs) to PC, thus increasing PUFA content of TGs, and impairing PC synthesis, hindering lipid droplet hydrolysis. Rs738409 is an established indicator of NAFLD risk with genome-wide significance demonstrated by conventional genotyping, bioinformatics, and novel natural language processing algorithms. These studies affirm rs738409 as an important indicator of histologically-confirmed steatosis, and a potent predictor of NASH risk [25], as rs738409 G-allele carriers are prone to liver decompensation, the development of HCC, and even death, especially in patients with advanced disease (i.e., F3 fibrosis or cirrhosis) [32]. On the other hand, *TM6SF2* encodes a regulatory protein of VLDL secretion and its variant, p.E167K (rs58542926), and depletes PUFAs, thus impeding VLDL synthesis. Rs58542926 has positive correlations with NAFLD risk, disease severity, and steatosis degree. This allele influences cirrhosis, and predisposes to HCC [25].

Finally, analysis of serum metabolomes of patients with NAFLD allowed to identify three distinct subtypes of NAFLD, based on their similarity to the metabolome profile of methionine adenosyltransferase 1a knockout (MAT1A-KO) mice, which have chronically low level of hepatic s-adenosylmethionine, and spontaneously developed NASH [33]. Although the different subtypes were equally represented in the NAFLD and NASH patients analyzed, which suggests that subtypes did not distinguish higher risk to develop NASH, the authors explained that s-adenosylmethionine therapy could be a better therapeutic approach for those patients with a subtype more similar to that of MAT1A-KO mice [33]. Furthermore, very recently, authors have shown that patients carrying a metabolomic signature similar to that of MAT1A-KO mice had a reduced risk of developing cardiovascular disease [34].

### 2.2. Carbohydrates Metabolism Disruption

Globally, dietary patterns have been changing over time, becoming highly enriched in carbohydrates, particularly monosaccharides and disaccharides, such as fructose and sucrose (composed of one glucose and one fructose molecule), respectively, and added sugars [35,36] (Figure 1). Long-term maintenance of these eating habits has been closely related to IR development and, consequently, to the onset of T2DM [37], which is one of the main comorbidities of NAFLD [38]. Indeed, NAFLD patients have more than a two-fold higher risk of incident T2DM than those without NAFLD [39]. Inversely, NAFLD is present in up to 75% of patients with T2DM [40]. Moreover, the prevalence of NASH and advanced fibrosis is increased among individuals with the coexistence of NAFLD and T2DM in comparison to non-diabetics with NAFLD [41,42]. Interestingly, there is a non-invasive method (i.e., OWLiver^®^DM2), based on a specific metabolomic profile, which is able to distinguish between steatosis and NASH in more general and multiethnic populations, including non-diabetic subjects and diabetic patients (both with and without controlled T2DM) [43]. Not surprisingly, liver-related complications are also more predominant in patients with NAFLD and T2DM [40]. Attending to this epidemiological data, a complex and bidirectional association between NAFLD and T2DM has been suggested, serving glycemic levels and IR as a potential nexus between both entities [20,44,45]. 

A chronic and excessive consumption of dietary carbohydrates and sugars cause plasma membrane accumulation of *sn*-1,2-DAG in myocytes, adipocytes, and hepatocytes, promoting the activation of the isoforms ε (in liver and adipose tissues) and θ (in skeletal muscle) of the protein kinase C (PKC) [46,47,48,49]. In turn, activated PKCε and PKCθ isoforms phosphorylate insulin receptor at Thr^1160^ and Ser^1101^/Ser^307^, respectively, impairing insulin downstream signals, and triggering IR [46,47,50].

#### 2.2.1. Glucose Metabolism

Postprandial glucose clearance is compromised by IR since both peripheral glucose uptake and insulin-mediated hepatic gluconeogenesis and glycogenolysis suppression fail, promoting DNL by two different molecular mechanisms [45,51]. Thus, elevated plasma glucose concentrations are redirected to the liver (i.e., glucose turnover), and partially uptaken via glucose transporter 2 (GLUT2) [45] (Figure 1). Then, the glucokinase (GCK)-mediated phosphorylation of glucose rapidly produces glucose 6-phosphate, which, in turn, is essential either for nuclear translocation or transactivation (by acetylation and O-GlcNAcylation post-translational modifications) of carbohydrate response element-binding protein (ChREBP) [52]. Once in the nucleus, ChREBP exerts transcriptional activity by binding to the conserved carbohydrate response element motif presented on promoters of the lipogenic genes such as *acetyl-CoA carboxylase (ACC), fatty acid synthase* (*FASN), steaoryl-CoA desaturase,* and *ELOVL fatty acid elongase 6* [53]. On the other hand, the impaired liver insulin response triggers a condition termed “selective hepatic IR”, which is characterizes by a zonation-dependent mechanism that perpetuates both gluconeogenesis and DNL processes [54]. In this regard, insulin receptor substrate (Irs) 1 is less abundantly expressed in the hepatic periportal zone, where the expression of Irs2 is downregulated, preventing insulin-mediated AKT activation, nuclear exclusion of the transcription factor Forkhead Box 01, and suppression of hepatic gluconeogenesis. The hepatic perivenous zone is enriched in Irs1, promoting activation and nuclear translocation of SREBP-1c vía AKT/mTORC1, and increasing the expression of key enzymes that regulate DNL [54,55]. In addition, IR in adipose tissue results in lipolysis exacerbation and elevated plasma FFAs levels, contributing to disease development and progression [56] (Figure 1).

#### 2.2.2. Fructose Metabolism

Unlike glucose, dietary fructose is primarily funneled, through portal circulation, into the liver (up to 70%), where GLUT2 and GLUT8 act as the major contributors to fructose uptake [21,57] (Figure 1). Once in the liver, fructose is rapidly phosphorylated by any of the two ketohexokinase splice variants (i.e., A and C) to fructose 1-phosphate (F1P), which promotes the release of GCK from inhibitory GCK regulatory protein, contributing to both glycolysis, and activation of the master transcriptional regulator of lipogenic genes, ChREBP [58]. Additionally, ChREBP can be also directly activated by fructose-derived metabolites [58]. Subsequently, F1P is metabolized by aldolase B in dihydroxyacetone and glyceraldehyde, which, in turn, participate in DNL as glycerol 3-phosphate and glyceraldehyde 3-phosphate, respectively [59]. Thus, glycerol 3-phosphate leads to TGs and lipoproteins synthesis, whereas glyceraldehyde 3-phosphate is used in mitochondrial acetyl-CoA production [59]. After being transported by the tricarboxylate transport system, cytoplasmic acetyl-CoA is not only a substrate for FAs and cholesterol synthesis, but also for both histone and liver X receptor (LXR) α acetylation, both increasing SREBP-1c expression, and indirectly regulating DNL [60,61,62]. Finally, F1P also serves as an activator of PPAR-γ coactivator 1 beta (PGC-1β) protein, simultaneously increasing the expression of SREBP-1c and ChREBP via direct binding-mediated transactivation [49,63].

Therefore, chronic hyperglycemia can trigger liver deleterious effects by promoting lipotoxicity and glucotoxicity [3] (Figure 1).

### 2.3. Immunologic System Distrubances

An inappropriate immune response is known to have a role in NAFLD progression. Although the exact mechanisms responsible for the development of NASH and liver cirrhosis or HCC are not completely elucidated, inflammation has shown to be implicated in these evolved stages of NAFLD [64]. 

#### 2.3.1. Triggers of Inflammation

Epithelial cells and innate immune cells are endowed with conserved pattern recognition receptors (PRRs), capable of sensing either damage-associated molecular patterns (DAMPs) or pathogen-associated molecular patterns (PAMPs) [65]. Upon binding of their ligands, different inflammatory pathways are activated, turning on transcription factors, such as NF-κB and interferon (IFN) regulatory factors (IRFs), and producing several inflammatory cytokines in order to respond to the insult, and restore tissue homeostasis [66]. Some well-studied PRRs are TLRs, present in many cells in the liver [67,68]. Some of these TLRs have been implicated in the pathogenesis of NAFLD and NASH, such as TLR2, which usually forms heterodimers with TLR6, and senses peptidoglycan; TLR4, which binds myeloid differentiation factor 2 protein (MD-2), and senses lipopolysaccharides (LPS); and the intracellular TLR9, implicated in sensing unmethylated DNA as the mitochondrial DNA (mtDNA) [69,70,71,72]. Nucleotide-binding oligomerization domain (NOD) family members can also sense DAMPs and PAMPs, and the activation of some of them can lead to inflammasome formation, such as the NOD-, LRR-, and pyrin domain-containing protein 3 (NLRP3) inflammasome, which has been reported to be implicated in NAFLD pathogenesis [73].

In NASH, such inflammation is initiated in the absence of pathogens, which is called sterile inflammation [74]. Once immune cells are activated, as the initiating stimulus persists, the inflammatory response becomes exacerbated and chronic, causing tissue remodeling and fibrosis. A source of concern in this field of research has been the identification of the pathogenic triggers of inflammation, promoting the transition from simple steatosis to NASH. More than 20 DAMPs, such as purine nucleotides, high-mobility group box 1, heat shock proteins, and nuclear and mtDNA or S100 proteins, have been described to activate PRRs, and originate an inflammatory response [74]. These inflammatory triggers may originate from within the liver or from other sites, such as adipose tissue or the intestinal tract (Figure 1).

##### Lipotoxicity

Among the pathogenic initiators from within the liver, lipotoxicity plays an important role. Lipotoxic molecules, as mentioned before, are able to directly initiate inflammatory signaling through pathways initiated by sensors, such as TLR4 or NLRP3 [69], leading to hepatocyte injury and death [11,75]. These injured hepatocytes release DAMPs to the medium, such as the sonic hedgehog ligand, in the form of soluble molecules [76]. In addition, injured hepatocytes can release extracellular vesicles (EVs), as evidenced by elevated levels of microvesicles found in experimental and human NASH [77]. EVs might contain lipids, miRNAs, DAMPs, and receptors contributing to the crosstalk of hepatocytes with immune cells and inflammation [77,78,79,80]. Cellular stress may also lead to apoptotic death of hepatocytes. Indeed, apoptosis levels have been correlated with NAFLD development [81]. Although this type of death is not highly pro-inflammatory, the engulfment of apoptotic bodies by KCs stimulates death ligand and cytokine expression [82]. Other lytic, more pro-inflammatory deaths have been also reported in NASH, such as ferroptosis, necroptosis, and pyroptosis [83,84]. These lytic deaths elicit strong inflammatory responses due to cell membrane permeabilization and the release of cellular components to the extracellular milieu, contributing to the recruitment of immune cells and the activation of HSCs [85] (Figure 1). 

##### Adipose Tissue-Derived Mediators

On the other hand, the relevance of adipose tissue inflammation in obesity and MetS is widely accepted [86,87] (Figure 1). Visceral and subcutaneous adipose tissue of NAFLD patients has an increased expression of genes that regulate inflammation, and VAT in these patients has shown greater proportions of pro-inflammatory CD11c^+^CD206^+^ and CCR2^+^ macrophages, which produce increased levels of inflammatory cytokines [88]. Evidence for the crosstalk between adipose tissue resident immune cells and the liver in NASH development is illustrated by a study exploring the effect of macrophage-containing VAT transplantation from donor mice to a murine NASH model (i.e., high cholesterol diet (HCD)). This setting revealed that obese-derived adipose tissue transplantation increases hepatic macrophage content compared with lean donor-transplanted mice, worsening liver damage. In addition, CD11c^+^ macrophages from adipose tissue of obese donors had greater expression of neutrophil chemoattractant molecules CXCL14 and CXCL16, suggesting also a role for neutrophil recruitment in this liver damage [89]. In addition, an accumulation of ectopic fat, including visceral obesity and fatty liver, leads to a dysfunction of the adipose tissue, with impaired production of adipocytokines, which, in turn, favor an increase in pro-inflammatory cytokines, such as tumor necrosis factor alpha (TNF-α) [90]. In this sense, a meta-analysis concluded that reduced levels of adiponectin, an adipocytokine known for its anti-inflammatory, anti-steatotic, and antifibrotic effects, are associated with progression to NASH [91]. On the other hand, another meta-analysis found that higher levels of circulating leptin were associated with increased severity of NAFLD [92]. In line with this, a study on NAFLD patients undergoing laparoscopic sleeve gastrectomy reported improved liver histology together with increased adiponectin levels and reduced serum levels of leptin and resistin [93].

##### Gut-Derived Mediators

The GLA is the bidirectional interaction between the gut and its microbiota and the liver. This crosstalk is established through the portal vein, which transports gut-derived molecules to the liver. Some of these molecules are PAMPs, such as the aforementioned LPS, as well as bacterial-derived metabolites generated through fermentative pathways [94], such as short-chain fatty acids (SCFAs) and alcohols or trimethylamine, among others.

The alteration of human gut microbiota (GM) (i.e., dysbiosis) partially contributes to the progression and onset of NAFLD, leading to a disruption of the GLA at different levels, such as the dysfunction of the epithelial layer [95]. As a result, there is an increase of gut permeability to microorganisms and bacterial-derived products that may reach the liver through the portal circulation, triggering the immune system, and promoting inflammation (Figure 1) [96]. 

There is recent evidence that bacterial-derived metabolites are involved in the pathogenesis of NAFLD. For example, SCFAs derived from the fermentation of dietary fiber serve as an energy source for colonocytes, enhancing the integrity of the intestinal epithelial barrier. SCFAs can also cross this barrier, and modulate the immune function, or exert anti-proliferative effects, in part by signaling through G protein-coupled receptors in multiple tissue sites [97].

On the other hand, alcohols have been suggested to contribute to NAFLD development. For example, ethanol can disrupt the GLA at different points, such as the epithelial barrier or GM composition itself, increasing microbial exposure and the pro-inflammatory environment of the liver. Accumulation of ethanol leads to intestinal inflammation, monocyte overproduction, and the production of TNF-α by macrophages, increasing intestinal permeability, translocation of bacterial products, and liver inflammation [98]. This ethanol has been suggested to be—at least partially—from a bacterial origin [99].

NAFLD has been associated to a decrease in human GM diversity [100,101], and to an alteration in its composition (Figure 1). For example, several studies have associated NAFLD to an increase in *Bacteroides* and *Escherichia* [102,103,104]. However, there are inconsistent, and even conflicting, results across studies aiming to characterize specific human GM signatures associated to NAFLD, in terms of taxonomic composition, function, and metabolic production. These contradictory findings can be attributed to multiple confounding factors, such as heterogeneous demographic variables of patient cohorts; spurious medical conditions added to NAFLD, such as obesity or IR; the definition and diagnostic criteria of NAFLD used in each study; endogenous or exogenous factors that influence the human GM; or a lack of standards in the laboratory and computational tools used to process and analyze the data [105,106]. 

#### 2.3.2. Role of Innate and Adaptive Immunity Components

##### Hepatocytes and Sinusoidal Endothelial Cells

Parenchymal hepatocytes are able to sense excessive levels of metabolites, DAMPs, and PAMPs through PRRs such as TLRs, cytoplasmic receptors such as the stimulator of IFN (STING), retinoic acid inducible gene-1 (RIG-I), and NOD family members, and initiate inflammatory events [65]. Of note, hepatocytes are the major source of LPS-binding protein (LBP), soluble CD14, and soluble MD-2 proteins, all of them required for LPS interaction with TLR4 and downstream signaling, as their expression is upregulated by interleukin (IL) 6, TNF-α, and other cytokines [107].

Hepatocytes also respond to the stimulation of cytokines by producing IL-6 and acute phase proteins, such as reactive C protein, which regulate the immune response, and kill bacteria [107,108]. Interestingly, hepatocytes are the main producers of complement proteins and their soluble regulators [107]. Accordingly, a Chinese study demonstrated that serum C3 levels are independently associated with a higher prevalence of NAFLD in an adult cohort [109]. Moreover, another study showed hepatic deposition of activated C3 and C4d in 74% of the NAFLD patients, but not in healthy liver subjects. This complement activation was, indeed, associated with apoptotic cells, hepatic neutrophil infiltration, as well as IL-8, IL-6, and IL-1β expression [110]. 

Similarly, sinusoidal endothelial cells (LSECs) also act as immune sentinels detecting PAMPs and DAMPs [111]. During NASH progression, LSECs acquire a pro-inflammatory phenotype characterized by overexpression of adhesion molecules at their surface, including intracellular adhesion molecule 1, vascular cell adhesion molecule 1, and vascular adhesion protein 1, as observed in mouse models of NASH and patients, contributing to leucocyte recruitment into the liver [112,113]. LSECs also produce a number of pro-inflammatory mediators in NASH, including CCL2, CCL5, and IL-6 [114].

##### Macrophages

Macrophages have been proposed to play a key role in NAFLD progression to NASH [115]. This idea is supported by the presence of portal macrophage infiltration early in NAFLD development, and its association with progressive disease [116]. The hepatic macrophage pool consists of liver-resident KCs and monocyte-derived macrophages [117]. In NAFLD, macrophages can be activated by multiple stimuli, such as lipids, oxidative stress, PAMPs, DAMPs, or cytokines [73,118,119,120].

M2 macrophages’ signature in liver biopsies from morbidly obese individuals seems to characterize patients with minimal hepatic steatosis as compared with those with severe steatosis [121]. Accordingly, several in vivo [121,122] and in vitro [123] studies have shown that pharmacological interventions polarizing M1 macrophages to an M2-predominant phenotype were associated with disease improvement [121,122,123]. Activated M1 KCs release pro-inflammatory cytokines, such as TNF-α, IL-6, and IL-1β, and chemokines (i.e., CXCL1, CXCL2, CCL2, and CCL5), affecting other liver-resident and immune cells, and mediating the recruitment of neutrophils and monocyte-derived macrophages [124]. 

The role of the two different subsets of macrophages in NAFLD development is dynamic. At the onset of a MCD-fed model, KCs seem to have a key role orchestrating damage and inflammation, as their depletion at this point attenuated the progression of NASH in vivo [125]. However, these cells were lost early in the natural course of the disease model followed by a robust CCR2- and CX3CR1-dependent infiltration of monocyte-derived macrophages [125,126]. Monocyte-derived macrophages are thought to have a pro-inflammatory phenotype, and seem to be functionally different from KCs. For instance, stimulation with palmitate showed to activate NADPH-oxidase-2-producing ROS through TLR4 signaling in monocyte-derived macrophages, but not in KCs, a mechanism that could generate greater liver injury [127]. Remmerie et al. showed that in a NASH murine model fed a MCD, two distinct types could be identified among the recruited macrophages: (i) macrophages which resembled KCs in their genetic expression; and (ii) macrophages similar to adipose tissue-derived macrophages, which expressed inflammatory cytokines and osteopontin [126], a recently proposed biomarker of NASH in patients’ serum [128].

##### Neutrophils

Peripheral blood neutrophil frequency is increased in patients with NASH compared to simple steatosis patients [129], having a neutrophils-to-lymphocytes ratio independent correlation with advanced inflammation and fibrosis in patients with NAFLD [130]. Neutrophil infiltration is also found in liver biopsies of NAFLD patients [116], as its recruitment is mediated by interleukins and chemokines and their ligands, such as C-X-C motif chemokine ligand 1 (CXCL1), which is upregulated in livers from NASH patients, but not in simple steatotic livers from obese individuals [131]. Neutrophils’ implication in NASH pathogenesis has been proved by neutrophil depletion in an HFD-fed mouse model, which improved metabolic parameters, steatosis, and inflammation in association with a decline in mice weight [132].

Molecules contained in neutrophils’ granules seem to have a role in neutrophil-caused damage in NASH. In this sense, mieloperoxidase has been found to be elevated in obese patients with NASH compared to simple steatosis [133]. This enzyme might directly cause hepatocyte injury, and activate HSCs, and its deletion in HFD-induced NASH decreases liver inflammation and fibrosis [134,135]. Similarly, elevated plasma levels of the neutrophils’ serine proteases elastase and proteinase 3 have been found in NAFLD and T2DM patients associated with NASH and fibrosis [136]. Neutrophil elastase has shown to have an effect on steatosis and inflammation as evidenced in a long-term Western diet-fed murine model of NASH, a role potentially associated with the ability of elastase to regulate the metabolism of hepatic ceramides [137]. Another neutrophil granule molecule, lipocalin-2, has been found elevated in serum and liver of both NASH patients and a high-fat high-cholesterol (HFHC) diet-fed ApoE^-/-^ murine model in association with hepatic neutrophil infiltration [138]. Experimental depletion of lipocalin-2 resulted in attenuation of hepatic injury, inflammation, and neutrophil infiltration, whereas chronic administration of this molecule exerted the opposite effects. Mechanistically, lipocalin-2 induced (C-X-C motif) receptor 2 (CXCR2) chemokine expression in neutrophils, facilitating their recruitment to the liver and crosstalk of these cells with hepatic macrophages [138].

Moreover, elevated levels of a neutrophil extracellular traps (NETs) marker have been found in the serum of patients with NASH, and NETs have been found in the livers from STAM mice (NASH induced by neonatal streptozotocin and high-fat diet) [139]. In vivo inhibiting of NET formation did not affect the development of steatosis, but decreased liver inflammation and HCC tumor growth [139].

##### Natural Killer and Innate Lymphoid Cells

Innate lymphoid cells (ILCs) are a heterogeneous family of non-T non-B lymphocytes strategically located on the surface of epitheliums. This family includes: natural killer (NK) cells, ILC1s, ILC2s, ILC3s, and lymphocytes tissue-inducer cells [140].

In studies with NASH patients, hepatic CXCL10 related to NK recruitment was elevated [141]. However, the frequency of hepatic and peripheral NK cells were not different between NASH patients and healthy controls, but the phenotype of CD56^bright^ and CD56^dim^ NK cells in the peripheral blood showed much higher expression of NKG2D [142]. Interestingly, in NASH patients with obesity, the number of hepatic NK cells was significantly increased compared with that in the healthy controls [143]. Nevertheless, the ability of intrahepatic NK cells to degranulate might be impaired in NAFLD patients [144].

Contrary, in glycine N-methyltransferase^−/−^ mice, which developed spontaneous progressive NAFLD, NK cells were activated, more TNF-related apoptosis-inducing ligand was expressed, and there was strong cytotoxic activity in the liver at early stages of disease [145]. However, a study in MCD-fed mice found increased conventional NK cells which prevented NASH progression to fibrosis by IFN-γ-dependent M1 polarization of macrophages, whereas a loss of hepatic ILC1s was found [146]. On the same line, another study found that increased recruitment of conventional NK cells into the liver resulted in a protective effect in progression to fibrosis through attenuated infiltration of monocyte-derived macrophages, particularly subsets skewed toward M2 [147]. Finally, in obese livers, it has been shown that NK cells might convert toward ILC-1-like cells, partially mediated by increased TGF-β. This could carry a reduction in cytotoxicity, which could be protective in the progression of NASH [144]. Of note, the protective or detrimental effects of NK cells and ILC1s depend on their environment and the stage of the disease. In this way, a NK-induced switch of phenotype in macrophages toward the M1 phenotype might worsen inflammation, and lead to steatohepatitis, but be protective against fibrogenesis in progression to cirrhosis.

Little is known yet about the role of ILC2 and ILC3 in NAFLD. However, their cytokine profile resembles that of the different T helper (Th) subsets, which have been widely studied. In this way, ILC2s are characterized by Th2 type cytokine production, such as IL-5 and IL-13, whereas ILC3s produce cytokines typical from Th17 cells (i.e., IL-17A and/or IL-22) [148].

##### B Lymphocytes

Oxidative stress causes molecular damage, generating oxidative stress-derived epitopes (OSEs), able to elicit both cellular and humoral adaptive immune responses [149]. NAFLD patients have higher hepatic infiltration of B cells in association with increased levels of circulating OSEs-directed IgG antibodies [150]. HFD-fed and high-fat high-carbohydrate-fed models showed increased B cell infiltration in livers with increased expression of IL-6 and TNF-α, and enhanced Th1 cell differentiation capacity [151,152]. Increased hepatic expression of B-cell activating factor (BAFF) seems to influence B cell activation in NASH, which precedes T cell responses. In addition, BAFF neutralization prevented liver plasma cell maturation, and decreased parenchymal damage and lobular inflammation in a NASH MCD-fed model [150]. These intrahepatic B cells seem to be pathogenic, since in vivo depletion of B2 cells reduced Th1 responses, and partially prevented NASH-related inflammation [150].

##### Conventional T Lymphocytes

CD4^+^ Th cells have been widely associated with NAFLD pathogenesis. In this way, a blockade of CD4^+^ T cells recruitment to the intestine and liver ameliorated hepatic inflammation and fibrosis while improving NASH-associated metabolic dysfunction in a western diet-induced NAFLD model [153]. On the other hand, a study in a MCD-induced NAFLD showed decreased intrahepatic CD4^+^, but not CD8^+^ T, cells in association with NAFLD-promoted HCC, a finding which could possibly occur due their activation and more extensive ROS production. In addition, IFN-γ and IL-17 production was increased in intrahepatic T lymphocytes [154]. When it comes to Th1 and Th2 implication in NAFLD, several studies consider NASH a Th1-polarized disease [155,156,157], whereas other studies have also observed a higher frequency of Th2 cells among circulating CD4^+^ T cells in patients with NAFLD [158,159]. In addition, the implication of Th17 cells and the production of IL-17 has been reported in multiple studies both in patients and mice [159,160,161], and its pathogenic role has been proved by inhibition of IL-17A/IL-17AR signaling, which protected mice from diet-induced liver steatosis and liver injury [160,162]. Similar effects were obtained when blocking differentiation of Th17 cells [163]. Additionally, IL-17 promotes not only pro-inflammatory cytokine production by macrophages, but also fibrosis through HSCs activation [164]. Accordingly, in an MCD-induced NAFLD model, two increases in Th17 cells were observed, one at the beginning of NASH development, and another at the NASH-fibrosis transition, whereas levels of Th22 cells peaked between the two expansions of Th17 cells [159].

Regarding Treg implication in NAFLD, the frequency of peripheral blood resting Tregs was decreased among CD4^+^ T cells in NASH, and to a lesser degree in simple steatosis [159]. On the other hand, hepatic Treg numbers seem to be increased in NASH [165]. In patients, peripheral blood Th17/resting Treg and Th2/resting Treg ratios are significantly increased in NASH versus simple steatosis [159]. This increased Th17/Treg ratio was also seen in mice HFD and MCD models, in which treatment shifting Th17/Treg imbalance towards a Treg dominance alleviated hepatic steatosis and inflammation [162,166].

On the other hand, infiltration of CD8^+^ cytotoxic T (Tc) cells is high in NAFLD patients in association with the stage of disease [116]. This hepatic infiltration of Tc cells is linked to obesity and IR, and promotes pro-inflammatory cytokines, as well as chemotactic molecules contributing to liver inflammation and progression to NASH [167,168]. Nevertheless, it has also been proposed that Tc-cells-derived perforin might exert immunomodulatory effects protecting from NASH development [169]. Finally, the memory phenotype of both CD4^+^ and CD8^+^ T-cells predominates above naive T-cells in the peripheral blood of NAFLD patients [158,170].

##### Innate-like T Cells

The frequency of intrahepatic and circulating NKT cells was measured in patients undergoing bariatric surgery, and was higher in those with moderate and severe steatosis than in those with mild steatosis [171]. Regarding iNKT (NKTs with TCR composed by invariant αβ chains and specially enriched in the liver), the frequencies in peripheral blood of activated CXCR3^+^ IFN-γ^+^ T-bet^+^ and IL-17A^+^ iNKT cells were also increased in NASH patients in comparison with those with simple steatosis or healthy controls [172]. In addition, in patients with NASH, the intrahepatic frequency of NKT cells was increased in more severe cases, in association with cirrhosis through HSCs activation [173,174]. Of interest, in these cases, NKT cells showed higher production of IL-4 [173]. In a CDAA-induced NASH model, the presence of iNKT cells was also necessary for the steatosis, steatohepatitis, and fibrosis [172,175]. In this case, the cytokine profile of iNKT cells showed two peaks, with NKT17 (IL-17^+^ iNKT and IL-22^+^ iNKT) and NKT10 (IL-10^+^-iNKT) increased during progression of steatosis, and NKT1/NKT2 (IFN-γ^+^ iNKT, IL-4^+^ iNKT and IL-13^+^ iNKT) elevated in progression to fibrosis. In addition, iNKT cells showed to have a role in the recruitment of macrophages and CD8^+^ T cells [172]. However, other studies found the opposite role of NKTs [176,177,178]. Thus, the role of NKT cells in NASH progression needs further exploration.

Mucosal-associated invariant T (MAIT) cells are characterized by a limited repertoire of TCR, with a Vα7.2^+^ chain (Vα19^+^ chain in mice) and predominantly Vβ 6 and Vβ chains [179]. These cells are highly enriched in livers, where they account for 20–50% of T cells [180]. The frequency of circulating MAIT cells was decreased in patients with NASH-related cirrhosis, but showed an activated phenotype, whereas they were also reduced in the liver, but accumulated within fibrotic septa. A study using a CCL14-induced model confirmed the role of MAIT cells in promoting myofibroblast proliferation, and their pro-inflammatory properties through the production of TNF-α [181]. Another study also found reduced levels of circulating MAIT cells, but in this case, they presented reduced production of IFN-γ and TNF-α, and increased IL-4, whereas, in this cohort, the presence of MAIT cells within the livers of NAFLD patients was higher, and correlated with NAFLD activity. An MCD-induced NASH model also showed enrichment of hepatic MAIT cells, which seemed to protect from inflammation by anti-inflammatory cytokine production and promotion of the M2 phenotype in macrophages [182]. Future research will hopefully aid in the clarification of MAIT cells’ role in NAFLD progression.

Finally, γδT cells, which recognize lipid antigens in a CD1d-dependent manner, are much more frequent in the liver than in other tissues, and hepatic γδT cells predominantly produce high levels of IL-17A. However, there is little evidence of the implication of these cells in NAFLD. Hepatic γδT17 cells are increased in HFD and high-fat high-carbohydrate diet-fed mice only in the presence of microbiota, contributing through IL-17A production to steatohepatitis, liver damage, and altered metabolism [183]. MCD-induced and HFD-diet followed by an ethanol binge steatohepatitis models showed increased hepatic γδT17 cells through CCR2 and CCR5 mediated recruitment, with distinct phenotypes to those in normal livers. Deletion, depletion, and targeted interruption of γδT cell recruitment protected against diet-induced steatohepatitis, and accelerated disease resolution [184].

## 3. Therapeutic Strategies According to the Disturbed Underlying Mechanism

### 3.1. Modulators of Lipid and Carbohydrate Metabolism

#### 3.1.1. Non-Pharmacological Treatments

At present, lifestyle-change-based therapies are recommended as the first-line therapy. The American Association for the Study of Liver Diseases stated that losing at least 3% to 5% of body weight could lead to remission of NAFLD in obese patients. In patients with histologically-proven NASH and with pooled liver biopsies before and after weight loss, improvement of all the histological features of NASH (i.e., steatosis, inflammation, ballooning, and fibrosis) was observed in those that achieved >−5% weight loss, whereas the greatest fibrosis resolution occurred in those with >−10% weight loss [185].

The latest EASL–EASD–EASO clinical practice guidelines recommend the Mediterranean diet as the diet of choice for all NAFLD patients [186]. The principal aspects of the Mediterranean diet are increased ω-3 and MUFAs intake, and decreased carbohydrate intake [28]. Increased physical activity has beneficial effects on NAFLD independently of weight loss. The EASL–EASD–EASO guidelines recommend 150 to 200 min/week of moderate-intensity aerobic physical activity in three to five sessions [186]. Besides, NAFLD cases possessing different *PNPLA3* alleles are more likely to benefit from lifestyle modifications than patients with other genotypes [28]. Exercise may also reduce methylation, and improve mRNA levels of mitochondrial genes, thus improving mitochondrial function [25].

However, long-term compliance with lifestyle modification is difficult to achieve and maintain in the target population. For this reason, we consider it important to introduce and disseminate the concept of liver rehabilitation, consisting in continuous and detailed programs of education for patients with this disorder, as well as a regular monitoring over time of their diet and physical activity. Probably, this triad approach (i.e., education + diet + exercise) will significantly increase the adherence to the only effective treatment so far.

On the other hand, a recent and prospective study with 180 patients who underwent hepatic biopsy before bariatric surgery, and both at 1 and 5 years afterwards, observed NASH resolution and fibrosis improvement in 84% and 70% of the patients, respectively, at the longest time [187]. These data suggest a great impact of bariatric surgery in hepatic histology improvement, although it is not considered a first-line therapy due to the associated high risk [188]. Currently, some endoscopic techniques which are not only less invasive, but also comprise less complications and costs, are being developed, accomplishing similar gastric restriction than that obtained with surgery. However, none of these methods are currently accepted as treatment for NASH. Despite this, there are some ongoing clinical trials on this field (NCT03426111 and NCT04060368) that may change the management of patients with NASH and obesity [188]. Preliminary results suggest that endoscopic sleeve gastroplasty could emerge as an effective and safe procedure, since therapeutic body weight reduction (i.e., >10%), improvement of biochemical parameters, and NAS score decrease were achieved [34,189].

#### 3.1.2. Peroxisome Proliferator-Activated Receptor Agonists

Thiazolidinediones (TZDs), including first-generation pioglitazone and rosiglitazone, are insulin-sensitizer agents that selectively activate the nuclear receptor PPAR-γ to both decrease gluconeogenesis, and increase glycolysis-related genes (Figure 1) [190,191]. Additionally, a pleiotropic effect of TZDs has been described by also attenuating mitochondrial pyruvate uptake that may condition the PPAR-γ agonistic potency of the first-generation TZDs [192]. Interestingly, the PPAR-γ agonist made lipid-induced M1-polarized macrophages switch to an M2-predominant phenotype *in vitro*, whereas treatment with rosiglitazone improved HFD-induced hepatic steatosis and lipid metabolism through reducing hepatic TLR4/NF-κB expression and M1-polarized KCs [123].

Despite pioglitazone being a 5-fold to 10-fold less potent PPAR-γ agonist than rosiglitazone, [192] it exerts more profound effects on NASH [193,194,195]. Thus, in the phase III PIVENS clinical trial (NCT00063622), non-diabetic patients with NASH received 30 mg of pioglitazone daily for 2 years, significantly improving the grade of hepatic steatosis, lobular inflammation, and hepatocellular ballooning, but not fibrosis, in comparison to the placebo group (Table 1). Moreover, levels of liver enzymes (i.e., AST and ALT) and IR were also reduced as consequence of pioglitazone treatment. Importantly, 47% of the subjects included in the PIVENS study reached NASH resolution [196]. Subsequently, a phase IV clinical trial (NCT00994682) obtained similar results in a cohort of patients with NASH and T2DM, reaching NASH resolution in 51% of the individuals assigned to the treated group (vs 19% in the placebo group), and improving of histological features after 3 years of pioglitazone administration at 45 mg/day (Table 1) [197]. Noteworthy, in this case, fibrosis was significantly reverted with pioglitazone, as observed in other clinical trials [194]. These results support the European and American clinical practice guidelines recommendation that pioglitazone may be prescribed to biopsy-proven NASH patients [198,199]. However, the use of pioglitazone is not largely extended in clinical practice for the treatment of NAFLD/NASH patients due to the associated adverse events (AEs), which include weight gain, fluid retention, increased risk of bone fractures, and possible bladder cancer [194,199,200].

On the other hand, long-term treatment of NASH patients (and 32% of them with T2DM) with 8 mg/day of rosiglitazone revealed an initial and maintained improvement of steatosis, whereas no effect was observed neither in the NASH nor fibrosis stage, as described in the phase II FLIRT2 clinical trial (NCT00492700) (Table 1) [195].

PPAR-α is a transcription factor highly expressed in hepatocytes, where it plays a crucial role through the activation of mitochondrial and peroxisomal fatty acid β-oxidation pathways. PPAR-α is particularly active during fasting, as it controls FA catabolism and ketogenesis, as well as the endocrine hormone fibroblast growth factor (FGF) 21. It has been shown to be activated by FFAs and eicosanoids, as well as phospholipids and endocannabinoids. Moreover, PPAR-α has anti-inflammatory properties, as it enhances FGF21 activity, and reduces NF-kB activity. However, though PPAR-α-targeted treatments have shown efficacy for NAFLD in preclinical studies, their effects in humans remain controversial [201].

Dual PPARα and PPARδ agonists (e.g., elafibranor) also stimulate mitochondrial and peroxisome β-oxidation, as well as Ω-oxidation [25]. Unfortunately, elafibranor phase III was terminated due to lack of compliance with primary surrogate efficacy endpoints (Table 1).

Various dual PPARα/γ agonists have shown promising results in human studies. Actually, four clinical trials (NCT03639623, NCT03617263, NCT04193982, and NCT05011305) are currently recruiting patients to evaluate saroglitazar effectiveness in different stages of NAFLD/NASH, and two phase II trials have already completed recruitment (NCT03061721 and NCT03863574), but no data has been published yet. Recently, a novel dual PPARα/γ agonist, called MD001, has been developed and tested in a *db*/*db* mice model, showing promising results by reducing hepatic steatosis through amelioration of hepatic TGs and FFAs, as well as glucose clearance and metabolism. Additionally, no remarkable AEs or toxicity have been detected after 2 months of daily administration of MD001 [202].

Pan-PPAR agonists are currently under evaluation. It was recently announced that lanifibranor met the primary endpoint of a reduction in steatosis activity fibrosis score, including NASH resolution without a worsening of fibrosis in a phase II clinical trial (NCT03008070) (Table 1); thus, being the first study that met both FDA and EMA regulatory endpoints for accelerated approval [203]. A lanifibranor phase III trial is recruiting (NCT04849728).

#### 3.1.3. Fibroblast Growth Factor Analogues

The precise mechanism of action of the hepatokine FGF21 is likely pleiotropic, involving glucose uptake by adipocytes and PPAR-α-dependent lipolysis, ultimately leading to improved insulin sensitivity, as it has been described in several diabetic in vivo models [204,205]. Consequently, this growth factor is in the spotlight as a potential and valuable therapeutic target for patients with NASH. In this regard, the pegylated FGF21 analogue, pegbelfermin, has been evaluated in a phase II clinical trial (NCT02413372) for patients with biopsy-proven NASH, where changes in the magnetic resonance imaging (MRI)-measured fat liver fraction were established as the primary outcome of the study. Thus, both the groups who received pegbelfermin at 10 mg/day and at 20 mg/week for 4 months showed a significant decrease in absolute fat fraction in comparison with the placebo group (Table 1). The drug was well tolerated, with only mild events: there were no reported deaths or discontinuations due to the AEs. However, the impact of pegbelfermin on liver histology was not assessed in this clinical trial [206]. For this reason, the phase II clinical trials FALCON1 (NCT03486899) and FALCON2 (NCT03486912) are currently underway in patients with NASH and liver fibrosis stage F3 and F4, respectively, in order to respond to this unmet need. Interestingly, preliminary results from the FALCON1 clinical trial have recently described the achievement of the primary endpoints (i.e., ≥1 stage fibrosis improvement without NASH worsening, or NASH improvement with no fibrosis worsening) after 24 weeks of administration of any dose of pegbelfermin (i.e., 10 mg, 20 mg, or 40 mg) compared to placebo group (14% of patients) [207]. However, no significant differences between treated arms were detected (i.e., 31% of patients at 10 mg, 24% of patients at 20 mg, and 27% of patients at 40 mg) [207]. Of note, this percentage of increase in primary endpoints responders was predicted by machine learning approaches [208]. Additionally, a post-hoc analysis of the FALCON1 clinical trial also showed a reducing effect of pegbelfermin on non-invasively-measured fibrosis, steatosis, inflammation, and ballooning with different duration [209]. Similarly, the FALCON2 clinical trial evidenced improvement of non-invasive determinations of fibrosis, steatosis, and inflammation in patients with compensated cirrhosis treated with any of the aforementioned doses of pegbelfermin during 48 weeks, in comparison to placebo group. Unfortunately, no statistical differences between the arms of the study were detected regarding the primary endpoint (i.e., fibrosis improvement without NASH worsening) [210].

The long-acting Fc-FGF21 fusion protein efruxifermin mimics the biological native activity and agonist potency of FGF21 on a receptor complex, constituting by the co-receptor *β*-Klotho and one of its cognate receptors. In the phase II BALANCED clinical trial (NCT03976401), patients with biopsy-proven NASH were given efruxifermin at three different daily doses (i.e., 28 mg, 50 mg, and 70 mg) for 4 months. Absolute hepatic fat fraction significantly decreased in a dose-dependent manner at 12 weeks in comparison to the placebo group, accomplishing the primary endpoint of the study. Of note, all patients treated with efruxifermin achieved ≥30% relative reduction of hepatic fat content at 12 weeks, and were eligible for an end-of-treatment liver biopsy (Table 1). This allowed evidencing NASH resolution without worsening fibrosis, and both fibrosis improvement (i.e., ≥1 stage) and NASH resolution in 48% and 28% of these responder patients, respectively [211]. Finally, a phase I clinical trial (NCT03298464), in which a single dose (240 mg) of the *β*-Klotho/fibroblast growth factor receptor 1 agonist NGM313 was evaluated, found a significant reduction in liver fat content and serum glycated hemoglobin (HbAc1), TGs, and low-density lipoprotein (LDL) levels after 36 days compared to the pioglitazone-treated group (Table 1) [212].

#### 3.1.4. De Novo Lipogenesis Inhibition

ACC catalyzes the conversion of acetyl-CoA to malonyl-CoA. Inhibitors of ACC lead to malonyl-CoA reduction, resulting in the downregulation of hepatic DNL (Figure 1), and mitochondrial β-oxidation [25]. The ACC-1 isoenzyme has a cytosolic localization, and is expressed in hepatocytes and adipocytes, whereas the ACC-2 isoenzyme is expressed on the mitochondrial surface of the liver, heart, and skeletal muscle [201].

Firsocostat is an inhibitor of hepatic ACC-1 and ACC-2, which led to a 29% reduction of liver fat content in 126 patients with NASH when given at a dose of 20 mg daily for 12 weeks in a phase II trial (NCT02856555) (Table 1) [201,213].

Furthermore, an ACC inhibitor, PF-05221304, and a DGAT2 inhibitor, PF-06865571, have been evaluated in combination. In two parallel, randomized, phase IIa studies in patients with NAFLD, authors reported a substantial reduction in hepatic steatosis with PF-05221304 and PF-06865571 vs. placebo, with all treatments generally well tolerated. The combination was effective in reducing the undesirable ACC inhibitor-mediated TG increases in serum, while preserving reductions in hepatic steatosis and transaminase concentrations (Table 1). A dose-dependent reduction in HbA1c was also noted in the overall population, with a greater effect in the T2DM subgroup at the individual PF-05221304 dose level. However, co-administration did not alter ACC inhibitor-induced increases in gamma-glutamyl transferase (GGT). The data presented in this study suggest that PF-05221304 and PF-06865571 co-administration is a potential option to counteract the limitations of PF-05221304 monotherapy, and to deliver greater clinical benefit than PF-06865571 or PF-05221304 alone, via a mechanistically grounded strategy [214].

FASN is one of the enzymes involved in DNL in the liver. In a phase II randomized, placebo-controlled trial, 99 patients with NASH were given 25 mg or 50 mg of TVB-2640 or placebo per day. Patients on the lower and higher dose showed a 9.6% and 25% reduction of liver fat content measured by MRI, respectively, compared to a 4.5% increase in the placebo group (Table 1). Safety monitoring revealed that this drug was well tolerated, without an increase in plasma TGs [202]. A phase II clinical trial evaluating the safety and efficacy on TVB-2640 in subjects with NASH (NCT03938246) concluded that TVB-2640 treatment was well tolerated, and improved metabolic, pro-inflammatory, and fibrotic markers [215].

Aramchol is an inhibitor of stearoyl-CoA desaturase, one of the key enzymes of DNL. Its usefulness in NASH has been evaluated in a phase IIb study (ARREST) where patients were randomized to 400 or 600 mg/day of aramchol or placebo. The 400 mg group showed a significant reduction of liver fat content, and a trend was observed in the 600 mg group compared with placebo. The resolution of NASH without worsening of fibrosis was more frequent in the 600 mg group than in the placebo group (Table 1). HbA1c was reduced, whereas no improvement in IR was observed. There were no severe AEs (NCT02279524) [216]. Currently, a phase III study is recruiting (NCT04104321). Moreover, aramchol treatment response in HIV-associated NAFLD has been also evaluated, but it did not reduce hepatic fat, or change body fat and muscle composition, determined by MRI-based assessment (NCT02684591) [217].

Others are targeting the production of pro-inflammatory lipids by inhibiting the enzyme 5-lipoxygenase (5-LOX), which catalyzes the lipooxygenation of arachidonic acid to leukotriene lipids [25]. The 5-LOX inhibitor MN-001 (tipelukast) has been tested in a phase II, open-labeled clinical trial, and is already completed (NCT02681055), but no results have been published so far.

#### 3.1.5. Treatments Targeting Cholesterol Metabolism

The key role that FC plays in the development of NASH has prompted research into the use of cholesterol-lowering treatments in these patients. Indeed, this was demonstrated in a multicenter cohort of 1201 patients where statins were protective against liver damage [3].

Furthermore, proprotein convertase subtilisin kexin type 9 (PCSK9) is a critical regulator of cholesterol metabolism primarily by inhibiting low-density lipoprotein receptor recycling, and thereby blocking the cellular uptake of low-density lipoprotein-cholesterol [218]. Animal studies have shown that downregulation of hepatic *Pcsk9* expression is associated with a pro-inflammatory phenotype during NASH development in middle-aged female mice [218]. However, human genetic studies did not confirm this evidence [219,220]. Thus, although PCSK9 inhibitors have been mainly developed for the treatment of hypercholesterolemia [218], early studies have shown that PCSK9 inhibitors could reduce steatosis biomarkers in some patients with both familiar hypercholesterolemia and NAFLD [221].

Ezetimibe, which is a drug that inhibits the absorption of FC in the small intestine, is able to decrease ALT levels, and suppress hepatic injury in NAFLD subjects. Two meta-analyses have studied the effect of this drug on NAFLD cases with variable BMIs, and reported that, although liver enzymes and NAFLD activity scores decreased, the results on fibrosis, inflammation, and steatosis were inconclusive [28].

BAs have also been linked to beneficial effects on glucose metabolism, mainly due to activation of TGR5 in enterocytes, leading to the release of glucagon-like peptide-1 (GLP-1), among others, which regulates food intake and glucose metabolism; whereas it has also been shown that BAs could lead to body weight reduction in mice. As discussed above, BAs are metabolized by GM. Therefore, changes in the composition of the GM can influence the pathways mediated by BAs, such as FXR signaling [222]. FXR agonists reduce lipotoxicity by stimulating cholesterol excretion, but also by promoting mitochondrial β-oxidation, and decreasing DNL. In phase II clinical trials (NCT01265498) and in an interim analysis of an ongoing phase III clinical trial (NCT02548351), obeticholic acid (OCA) reduced hepatic steatosis, inflammation, and fibrosis compared to placebo (Table 1) [25]. The FLINT study analyzed the effects of the semisynthetic BA, OCA, at a dose of 25 mg/day in patients with non-cirrhotic, non-alcoholic steatohepatitis compared to placebo for 72 weeks. The primary outcome was improvement in liver histology, defined as a decrease in NAFLD activity score by at least two points without worsening of fibrosis from baseline to the end of treatment. Therefore, OCA met the primary outcome in 46% of patients compared with 21% of patients in the placebo group (Table 1) [223]. However, OCA has been observed to increase plasmatic lipid concentration, and cause pruritus [222]. Norursodeoxycholic acid is another BA derivative under evaluation in a phase IIb trial in patients with NASH (NCT05083390).

There are other non-steroidal FXR agonists. Px-104 (NCT01999101), EYP 001 (NCT03976687, NCT03812029), cilofexor (NCT02781584, NCT03987074, NCT02854605), EDP-305 (NCT02918929, NCT04378010, NCT03421431), and tropifexor (NCT02855164) are being studied in phase I–IIa trials in patients with NASH. From them, only results for cilofexor (NCT03987074), EDP-305 (NCT03421431), and tropifexor (NCT02855164) have been released. In the case of cilofexor, the treatment with 100 mg for 24 weeks was well-tolerated, and provided significant reductions in hepatic steatosis, liver biochemistry, and serum BAs in patients with NASH [224,225].

Finally, due to low efficacy and AEs observed in trials, huge efforts towards the investigation of the effect of combination therapies are being performed. In this sense, the ATLAS trial, a phase II clinical trial (NCT03449446), evaluated the safety and efficacy of monotherapy and dual combination regimens of cilofexor 30 mg, firsocostat 20 mg, and selonsertib (an authopagy inhibitor) 18 mg in patients with advanced fibrosis, including those with NASH-related cirrhosis. In this study, a higher percentage of patients in the combination therapy group (cilofexor and firsocostat) achieved more than 1 stage improvement in fibrosis without worsening of NASH after 48 weeks of treatment compared with the placebo group (20.9% vs. 10.5%, *p* = 0.17) (Table 1) [201].

### 3.2. Antihyperglycemic Drugs

#### 3.2.1. Metformin and Dipeptidyl Peptidase 4 Inhibitors

Metformin is the first-line treatment for patients with T2DM, although it has been described that this biguanide has no impact on NASH histological endpoints, and, therefore, the use of metformin is not recommended for these patients [226]. Likewise, no data about the effects of dipeptidyl peptidase 4 (DPP4) inhibitors on liver histology of NAFLD patients is available, despite vildagliptin being shown to reduce MRI-measured liver fat content [227].

#### 3.2.2. Glucagon-like Peptide-1 Receptor Agonists

GLP-1 is a gut-derived incretin hormone capable of stimulating insulin secretion, and regulating glucose homeostasis by binding to its cognate receptor, glucagon-like peptide-1 receptor, which has been found in many tissues, including the pancreas, brain, and, more controversially, in the liver (detected in primary hepatocytes in vitro and cell lines) [228]. In this regard, different glucagon-like peptide-1 receptor agonists (GLP1-RAs) mimicking the action of GLP-1 have been developed and approved for the FDA to treat patients with T2DM, and their therapeutic effects have also been slightly explored in patients with NASH [193,226]. Importantly, the modified chemical structure of GLP1-RAs confers more resistance to enzymatic degradation by DPP4, and prolonged action on hepatic gluconeogenesis, glycogen synthesis, and glycolysis (Figure 1) [229,230]. These have already shown promising effects in many preclinical models of NAFLD and NASH [231]. Among GLP1-RAs, liraglutide has obtained the most compelling evidence so far about the potential impact of GLP1-RAs in NASH treatment due to the phase II LEAN clinical trial (NCT01237119). Thus, administration of liraglutide (1.8 mg/day) for 1 year to patients with biopsy-proven NASH resulted in a resolution of NASH with no worsening of fibrosis in 39% of the patients assigned to this arm in comparison with the 9% in the placebo group. Interestingly, a similar proportion of NASH resolution was observed in patients with and without T2DM (i.e., 38% and 40%, respectively) (Table 1). Furthermore, a reduction of body weight and BMI, an improvement of physical activity, and no severe AEs were associated with liraglutide treatment [232]. The longer acting GLP1-RA semaglutide has also been evaluated for subjects with biopsy-confirmed NASH. Recently, results from a phase II clinical trial (NCT02970942) highlight that subcutaneous administration of semaglutide once a day achieved NASH resolution in a dose-dependent manner (Table 1). Of note, this trial also observed improvement of fibrosis stage in 43% of patients with F2-F3, although it did not reach significant differences with respect to the placebo group [233]. Importantly, this trial was prompted by the beneficial impact of semaglutide over other GLP1-RAs observed in a series of phase III clinical trials (i.e., SUSTAIN trials), where reduced HbAc1, body weight, and cardiovascular risk were reported [234,235,236,237,238,239,240,241,242]. Although another study, in which semaglutide at 0.4 mg/day was administered for 72 weeks to patients with NAFLD, could not reach the primary endpoint (i.e., differences in liver stiffness), again, promising and interesting results emerged, including long-term maintained reduction in liver fat ≥30% in 70% of the semaglutide group, body weight loss, and liver enzymes normalization [243]. Currently, an ongoing phase II clinical trial (NCT03987451) is evaluating the therapeutic effects of semaglutide weekly administered to patients with NASH and compensated cirrhosis, with histological improvement in liver fibrosis as the primary outcome of the study. Finally, tirzepatide and cotadutide are two dual GLP1-RAs with gastric inhibitory polypeptide receptor activity and glucagon activity, respectively, which are being tested in phase II clinical trials for NASH patients (NCT04166773 and NCT04019561, respectively). Interestingly, in post-hoc analysis, both tirzepatide and cotadutide (NCT03131687 and NCT03235050, respectively) have already shown to improve some NASH-related biomarkers (Table 1) [244,245].

#### 3.2.3. Sodium Glucose Co-Transporter 2 Inhibitors

Similar to GLP1-RAs, sodium glucose co-transporter 2 inhibitors (SGLT2i) have become one of the second-line therapeutic options for the management of T2DM [246]. These compounds boost renal excretion of glucose by lowering kidney reabsorption, ultimately resulting in reduced circulating levels of glucose, and inhibition of both ChREBP and SREBP-1c activity [205,247]. As recently described, several studies report beneficial effects of SGLT2i in NASH in vivo models, such as attenuation of hepatic steatosis, inflammation, and fibrosis, as well as prevention of both NASH and NASH-related HCC development [248,249,250,251]. Regarding human clinical trials, only few relatively small studies have been conducted with SGLT2i in NAFLD/NASH patients, making it difficult to draw robust conclusions. Nevertheless, most of them have reported improvement of plasma aminotransferase levels, particularly ALT, with the SGLT2i emplaglifozin [252,253,254], dapaglifozin [255], canaglifozin [256], and ipraglifozin [257,258,259,260]. Unfortunately, none of these studies evaluated histological outcomes as the primary endpoint. Interestingly, the E-LIFT (NCT02686476) clinical trial evaluated the MRI-measured liver fat content reduction after administration of empagliflozin (10 mg/day) for 5 months in comparison to the control group (Table 1) [253]. This effect was also obtained with ipraglizofin (50 mg/day) in a Japanese cohort of patients, where the liver-to-spleen ratio was measured by abdominal computed tomography scans, detecting a significant increase of this ratio after 3 months of treatment with the SGLT2i [256]. Importantly, a recent and open-label pilot study (NCT02964715) provided primary histological evidence about the therapeutic relevance of empaglifozin for biopsy-proven NASH patients with T2DM. Thus, steatosis and hepatocyte ballooning improved after prescription of emplaglizofin (25 mg/day) for 6 months in the majority of subjects, and nearly half of them reached NASH and fibrosis resolution (Table 1) [261]. In line with this, an ongoing phase III clinical trial (NCT03723252) is evaluating the impact of dapaglifozin in patients with NASH, and with or without T2DM on liver histological features improvement and NASH resolution. Lastly, the dual SGLT1/2 inhibitor, licoglifozin, seems to be equally potent as empaglifozin for patients with T2DM and chronic heart failure [262], and is currently under investigation to treat NASH and liver fibrosis (NCT04065841).

### 3.3. Immune-Related Targets

#### 3.3.1. TLR4 Inhibitors

Different strategies have been probed in NASH patients to attenuate TLR4 activation (Figure 1). On the one hand, a phase II trial studied safety and potential efficacy of two different doses (5 mg and 10 mg, twice daily administered) of JKB121, a long-acting small molecule which acts as a weak antagonist of the TLR-4 receptor, in 65 patients with NASH (NCT02442687). This molecule had already proven its efficacy in an MCD-induced rat model of NAFLD, and, in vitro, it showed to reduce the LPS-induced release of inflammatory cytokines, and deactivate and inhibit HSCs proliferation and collagen expression. However, in this clinical trial, notable improvement in liver fat content, ALT, and fibrosis-4 index was observed in the placebo group at week 24, whereas JKB-121 did not further improve the response rate in patients with NASH compared to placebo (Table 1) [263]. On the other hand, a phase ll study addressed the safety and preliminary efficacy of an oral formulation containing anti-LPS polyclonal antibodies, named IMM-124E, vs. placebo for NASH treatment (NCT02316717). One-hundred and thirty-three patients were randomized into three groups: placebo, IMM-124E 600 mg 3 times/day, and IMM-124E 1200 mg three times/day, and continued treatment during 24 weeks. Treatment was well tolerated, and both IMM-124 arms showed improved ALT and AST levels, whereas the higher dose IMM-124 group also showed a decrease of serum cytokeratin-18 compared to the placebo arm. However, no improvement in hepatic steatosis was observed (Table 1).

#### 3.3.2. CCR2/CCR5 Antagonists

Another strategy has been preventing immune cell recruitment into the liver. Thus, treatment of NASH models by using cenicriviroc, an oral dual chemokine receptor CCR2/CCR5 antagonist, reduced infiltration of monocyte-derived macrophages, and ameliorated histological NASH activity and hepatic fibrosis [264]. For this purpose, it was evaluated in the CENTAUR study (NCT02217475), a phase IIb clinical trial in 289 patients. After 1 year of treatment with 150 mg/day vs. placebo, twice as many subjects achieved improvement in fibrosis and no worsening NASH compared with placebo (Table 1) [265]. These results, led to a phase III trial to confirm the efficacy and safety of this drug for the treatment of liver fibrosis in 1997 NASH patients (NCT03028740). However, the trial was terminated early due to the lack of efficacy of cenicriviroc in the first part of the study, in which the study group was expected to improve fibrosis in at least one stage, and present no worsening of NASH at month 12 (Table 1).

### 3.4. Microbiome-Targeted Therapy

#### 3.4.1. Prebiotics, Probiotics, and Synbiotics

These therapies consist of: (i) non-pathogenic microorganisms with health benefits to the host, mainly *Bifidobacterium*, *Lactobacillus*, and *Streptococcus* (i.e., probiotics); (ii) indigestible, fermentable substrates that promote the growth of probiotics, such as fructooligosaccharide (FOS) or inulin (i.e., prebiotics); and (iii) a combination of the previous two. Interestingly, they have been proposed in recent clinical studies as a therapeutic mechanism to drive the human GM associated to a NAFLD phenotype towards a homeostatic state (Figure 1) [266]. One of the first clinical trials (NCT03434860) using probiotic-based microbiome-targeted therapies (MTT) supplemented 58 T2DM and NAFLD patients with 14 strains from the bacterial genera *Bifidobacterium*, *Lactobacillus*, *Lactococus*, *Propionibacterium*, and *Acetobacter*. A decrease of fatty liver index (FLI), serum AST and GGT, and TNF-α/IL-6 levels were observed in the treatment group (Table 1) [267]. Nonetheless, in a posterior study (IRCT201410052394N13), 89 NAFLD patients were supplemented in a phase II trial either with five bacterial species of *Lactobacillus* and *Bifidobacterium* (i.e., *L. acidophilus*, *L. casei*, *L. rhamnosus*, *B. breve*, and *B. longum*), or with FOS. Both treatments significantly decreased serum concentrations of leptin, insulin, as well as IR compared to the control group. However, plasma glucose decreased only in the prebiotic group, questioning the relevance of probiotics in this clinical case (Table 1) [268].

A synbiotic-based MTT supplemented 52 NAFLD patients with Protexin in a phase II trial (NCT01791959) which contained seven bacterial species (i.e., *L. acidophilus*, *L. bulgaricus*, *L. casei*, *L. rhamnosus*, *B. breve*, *B. longum*, and *S. thermophilus*) and FOS. ALT decreased significantly in both the treatment and control groups (Table 1). However, the significance of this reduction was one order of magnitude lower in the treatment group, which suggested a superiority of the synbiotic supplementation for the treatment of NAFLD. Additionally, it was found that this effect is at least partially due to the reduction of NF-κB and TNF-α [269]. Another study supplemented 60 NAFLD patients in another phase II trial (IRCT201111082709N22) with Protexin and FOS, adding vitamin E to some of the study groups. The treatment with and without vitamin E also lowered ALT, leptin, plasma glucose, insulin, TGs, cholesterol, and LDL, observing a boosting effect of the vitamin in the comparison of some of these parameters (Table 1) [270].

Clinical trials with single-species probiotics have also been utilized lately. For example, a study supplemented 102 NAFLD patients with a synbiotic yogurt containing *B. animalis* and inulin (IRCT2017020932417N2). The treatment significantly reduced steatosis on abdominal ultrasound, serum ALT, AST, and GGT (Table 1) [271]. A more recent study (NCT01680640) also administered *B. animalis* and FOS to 104 NAFLD patients. In this case, their fecal samples were observed to have higher proportions of *Bifidobacterium* and *Faecalibacterium*, and lower proportions of *Oscillibacter* and *Allistipes*. However, these compositional changes in the microbiome were not associated to a reduction of liver fat content or fibrosis markers (Table 1) [272].

Another clinical study supplemented 138 NAFLD patients with Familact, which contained seven bacterial species (i.e., *L. acidophilus*, *L. bulgaricus*, *L. casei*, *L. rhamnosus*, *B. breve*, *B. longum*, and *S. thermophilus*) and FOS. All patients were also administered sitagliptin. The synbiotic treatment significantly reduced BMI, plasma glucose, ALT, AST, cholesterol, and TGs (Table 1) [273]. However, a previous similar study (IRCT2013122811763N15) with 80 NAFLD patients supplemented with the same symbiotic, but with no sitagliptin, did not output changes in ALT or AST levels, showing only a significantly reduced steatosis on abdominal ultrasound [274]. This could mean that sitagliptin partially behaved as a confounding factor in the first study, as mentioned above. The lack of direct involvement of the GM in this type of MTTs can also be observed in another phase II study (IRCT201301223140N6) that supplemented 75 NAFLD patients with a capsule containing *L. acidophilus* and *B. longum*, coupled with inulin, in different study groups. The prebiotic, probiotic, and synbiotic treatment lowered ALT and BMI when compared to control, with no differences between them (Table 1) [275].

In conclusion, several clinical trials have examined the use of non-fecal microbiota transplantation MTT in NAFLD, including some of the above, but yielded mixed results. In fact, probiotics have previously been reported not to modify GM composition in many clinical trials [276]. These results reassess the idea of abandoning GM taxonomical signatures as a potential biomarker to diagnose NAFLD.

#### 3.4.2. Fecal Microbiota Transplantation

Fecal microbiota transplantation (FMT) consists in transferring the human GM from the stool of a healthy individual to the GI of a patient with an altered one [266]. Clinical trials based on this therapeutic strategy for patients with NAFLD are scarce across the literature. One of these studies was performed by Craven et al. in 2020 [277]. In this phase II trial (NCT02496390), 21 NAFLD patients were subject to a FMT from three lean and healthy donors. Specifically, the patients were randomized to an either allogenic or an autologous FMT delivered by using an endoscope to the distal duodenum in a 3:1 ratio. The authors observed that allogenic FMT did not decrease IR or hepatic fat fraction assessed by magnetic resonance. However, half of the patients with elevated small intestinal permeability at baseline had a significant reduction 6 weeks after allogenic transplant. These patients experienced an almost significant increase in GM diversity 6 weeks after the treatment (Table 1), suggesting that FMT could prevent complications of increased intestinal permeability. However, they had a lack of changes in specific bacterial taxa, which could be due to FMT being administered into the duodenum, and GM analysis limited to stool taxa. This poses a limitation in the study, as it has been shown that changes in the small intestinal bacteria are not reflected by changes in fecal microbial diversity [278].

Another recent study performed a clinical trial with another cohort of 21 NAFLD patients that were subject to FMT from lean, vegan donors in allogenic and autologous transplants. Allogenic FMT altered human GM composition, increasing the abundance of *Ruminococcus*, *E. hallii*, *F. prausnitzii*, and *P. copri*, and increased the plasma concentration of the aminoacids isoleucine and phenylacetylglutamine. The procedure also led to changes in the genic expression involved in hepatic inflammation or lipid metabolism. Specifically, the expression of *ARHGAP18*, a protective gene that maintains endothelial cell alignment, increased after the treatment (Table 1), whereas the expression of *GGT* and *ALT* decreased [279]. Aside from these two studies, the impact of FMT on patients with NAFLD is being researched on participants in India (NCT04594954) and in the Netherlands (NCT04465032). There are also at least three discontinued studies (NCT02469272, NCT03803540, and NCT02721264), and two phase II trials (one in Spain, and one in the US) that have not yet commenced (NCT03803540 and NCT04371653, respectively). Of note, these last two clinical trials propose an interesting and novel approach in this field, administrating fecal microbiota in oral capsules. Although both studies will analyze the diversity in the microbiota profile, the Spanish clinical trial, led by our group, is the only one, so far, that plans to evaluate histological resolution of NASH with FMT capsules, and includes an innovative lead-in phase in order to control and mitigate the impact of lifestyle changes.

Other clinical trials using FMT from lean donors to obese patients with MetS, but without NAFLD, have reported a temporal increase of insulin sensitivity. This effect was associated with changes in GM, such as butyrate-producing bacteria, plasma metabolites, and glucose metabolism [278,280]. Interestingly, the recipient and donor strains were not closely associated in either of the studies, coexisting for a few months after the treatment, as was evaluated later [281]. As NAFLD is commonly associated with IR, these results suggest that FMT could be partially efficient in the management of the disease, as response to the treatment is modulated by differences in fecal GM composition. Another study discovered that FMT from metabolically compromised obese donors temporarily worsens insulin sensitivity in recipients with MetS, and, at the same time, increases insulin sensitivity in recipients from healthy post-gastric bypass donors [282]. And finally, a more recent clinical trial showed that changes in GM composition and BAs profiles after FMT were not associated with an obesity reversal in the treated patients [283]. In this work, no differences were observed in GLP-1 or BMI either. All of these results turn the effects of FMT on digestive pathologies into an issue to be clarified.

#### 3.4.3. Potential Intervention with Bacteriophages

Bacteriophages have arisen as a promising novel approach for the intervention of dysbiosis in the context of liver diseases, since they can target a certain strain of bacteria specifically without disturbing the entire microbiota [284]. Studies in alcoholic steatohepatitis have explored the effects of targeting patient-derived cytolytic *E. faecalis* transferred to mice, by treating them with highly specific bacteriophages. This strategy has shown to protect from ethanol-induced liver injury in the presence of this strain in vivo [285,286].

Although, as previously mentioned, different studies in NAFLD show inconsistent abnormalities in GM, a study by Yuan et al. found *K. pneumoniae* with high alcohol productivity within the intestinal microbiota of patients with NAFLD in greater proportions than in lean subjects [287], a finding which has been further corroborated in a cohort of 117 NAFLD patients with small intestinal bacterial overgrowth [288]. The pathogenicity of these bacteria in NAFLD was proven by FMT or gavage administration of these *K. pneumoniae* isolated from NASH patients to mice, which led to endogenous alcohol production, mitochondrial dysfunction, intestinal barrier damage, increased Th17 cells, and liver inflammation, accelerating NAFLD development. Interestingly, these effects were prevented when fecal microbiota was pre-treated with a bacteriophage, a finding that paves the way for future clinical trials with bacteriophages for NAFLD treatment.

**Table 1:** Completed clinical trials in NAFLD.

Identifier	Design	Drug (Mechanism)	Patients (n)	Patients’ Profile				Drug Administration		Primary Outcome	Main Outcomes	Refs
				Lipid Profile	Glucidic Profile	Immunologic Profile	Microbiotic Profile	Dose	Period			
Drugs targeting lipids and carbohydrates metabolism												
NCT00063622 (PIVENS)	Phase III	Pioglitazone (PPARγ agonist)	Non-diabetic patients with biopsy-proven NASH (247)	TGs: 165 ± 93 mg/dL TChol: 196 ± 39 mg/dL in all patients at baseline	Glucose: 94 ± 13 mg/dL HOMA-IR: 5.2 ± 4.3 in all patients at baseline	Hepatic inflammation	NA	Pioglitazone: 30 mg	Once daily	1.Improvement in NAFLD activity defined by change in standardized scoring of liver biopsies at 96 weeks	Significant improvement of histological features, excluding fibrosis, in comparison to PBO group. 47% of the subjects reached NASH resolution	[196]
								PBO	Once daily			
NCT00994682 (UTHSCSA NASH Trial)	Phase IV	Pioglitazone (PPARγ agonist)	Biopsy-proven NASH and pre-diabetes or T2DM patients (101)	FFA: 0.49 ± 0.18 mmol/L TGs: 224 ± 171 mg/dL TChol: 187 ± 46 In pioglitazone group	T2DM: 48% Glucose: 124 ± 29 HbA1c: 5.7 ± 0.5 (in non-T2DM patients) and 7.1 ± 0.9 (in T2DM patients) Insulin: 15 ± 11 µU/mL in pioglitazone group	Hepatic inflammation Neutrophils count: ≥1500/mm^3^ Platelets: ≥ 100,000/mm^3^ in all patients	NA	Pioglitazone: 30 mg (if tolerated 45 mg)	Once daily, 8 weeks	1. ≥2-point reduction in NAS (in at least two different histological categories) without worsening of fibrosis at 18 months	58% of patients assigned to pioglitazone achieved the primary outcome, whereas 51% had NASH resolution. Pioglitazone improved individual histological scores, including the fibrosis score, and insulin sensitivity.	[197]
								PBO	Once daily			
								Open label pioglitazone (all patients)	Once daily for an additional 18 months			
NCT00492700 (FLIRT 2)	Phase II (Extension phase)	Rosiglitazone (PPARγ agonist)	Biopsy-proven NASH with increased transaminase values (53)	TGs: 1.5 ± 1.1 mmol/L HDL: 1.2 ± 0.5 in treated group	T2DM: 24% Glucose: 5.5 ± 2 mmol/L Insulin: 13.5 ± 8.5 µUI/L HOMA: 3.3 ± 3.4 in treated group	Hepatic inflammation	NA	Rosiglitazone: 8 mg (4 mg the 1st month)	Once daily	1.Reduction in steatosis ≥30%	No improvement in the NAS score and histological features after 2 additional years of rosiglitazone administration	[195]
								PBO	Once daily			
NCT02704403 (RESOLVE-IT)	Phase III	Elafibranor (Dual PPARα/δ agonists)	Biopsy-proven NASH patients with BMI ≤ 45 kg/m^2^ (2157)	NA	49.6% T2DM	Hepatic inflammation	NA	Elafibranor: 120 mg	Once daily	1.Resolution of NASH without worsening of fibrosis. 2. Long-term outcome composed of all-cause mortality, cirrhosis, and liver-related clinical outcomes	Terminated, not accomplished	NA
								PBO	Once daily			
NCT03008070 (NATIVE)	Phase II	Lanifibranor (Pan-PPAR agonists)	Biopsy-proven NASH patients with BMI < 45 kg/m^2^ (247)	Tchol: 1.2 ± 0.3 mmol/L TGs: 2.0 ± 0.9 mmol/L	HbA1c: ≤8.5% Glucose: <10 mmol/L Insulin: 246.9 ± 260.7 pmol/L	Hepatic inflammation	NA	Lanifibranor: 800 mg	Once daily	1. SAF-A decrease of at least 2 points with no worsening of the CRN-F	SAF-A decrease of at least 2 points with no worsening of CRN-F for 1200 mg dose RR = 1.82 (95% CI 1.24, 2.4)	[203]
								Lanifibranor: 1200 mg	Once daily			
								PBO	Once daily			
NCT02413372	Phase II	Pegbelfermin (FGF21 analogue)	Biopsy-proven NASH with BMI ≥ 25 kg/m^2^ (184)	TGs: 187 ± 55 mg/dL HDL-C: 45 ± 12 mg/dL LDL-C: 120 ± 36 mg/dL	HbA1c: 6.2 ± 1.1%	Hepatic inflammation	NA	Pegbelfermin: 10 mg	Daily	1. Mean change in percent hepatic fat fraction by MRI	Significant decrease in absolute hepatic fat fraction in 10 mg/day and 20 mg/week groups compared with PBO group	[206]
								Pegbelfermin: 20 mg	Weekly			
								PBO	Daily			
NCT03976401	Phase II	Efruxifermin (Fc-FGF21 fusion protein)	Biopsy-proven NASH with BMI ≥ 25 kg/m^2^ and confirmation of ≥10% liver fat content on MRI-PDFF (80)	TGs: 180.0 ± 99.0 mg/dL TChol: 175.7 ± 45.1 mg/dL Apo B: 94.7 ± 27.8 mg/dL Apo C3: 9.4 ± 4.9 mg/dL	HbA1c: 6.23 ± 1.2% Glucose: 134.8 ± 66.2 mg/dL HOMA-IR: 14.1 ± 12.3	Hepatic inflammation	NA	Efruxifermin: 28 mg	Once weekly	1. Change from baseline in hepatic fat fraction assessed by MRI-PDFF	All efruxifermin-treated patients achieved ≥30%, and 88% achieved ≥50%, relative reduction in liver fat	[211]
								Efruxifermin: 50 mg	Once weekly			
								Efruxifermin: 70 mg	Once weekly			
								PBO	Once weekly			
NCT03298464	Phase I	NGM313 (β-Klotho/FGFR1 agonist)	Insulin resistant and obese patients (i.e., BMI: 30–43 kg/m^2^) with both increased liver fat and normal ECG readings (25)	NA	NA	NA	NA	NGM313: 240 mg	Single dose	1. Evaluation of whole body insulin sensitivity measured as insulin sensitivity index (M and Si) following intravenous insulin administration	Significant reductions in LFC (measured by MRI-PDFF), HbA1c, TGs, and LDL-C; and an increase in HDL-C, in NGM313-treated group	[212]
								Pioglitazone: 45 mg	Daily			
NCT02856555	Phase II	Firsocostat (ACC inhibitor)	MRI-PDFF ≥8% Liver stiffness by MRE >2.5 kPa (126)	TGs: 160 (125, 201) mg/dL Tchol: 179 (152, 203) mg/dL	HbA1c: 6.5 (5.8, 7.8) % Glucose: 117 (97, 149) mg/dL HOMA-IR: 8.7 (5.3, 13.0) Insulin: 26.1 (17.0, 48.9) µIU/ml	NA	NA	Firsocostat: 5 mg	Once daily	1.Percentage of participants experiencing treatment-emergent adverse events	AEs were experienced by 71% of patients receiving GS-0976, and by 62% with PBO	[213]
								Firsocostat: 20 mg	Once daily			
								PBO	Once daily			
NCT03776175	Phase II	PF-05221304 (ACC inhibitor) PF-06865571 (DGAT2 inhibitor)	Metabolic syndrome (99)	TGs: 175.3 ± 66.8 mg/dL Tchol: 194.5 ± 36.6 mg/dL Apo C3: 13.9 ± 7.0 mg/dL	HbA1c: 5.8 ± 1% Glucose ≥100 mg/dL	NA	NA	PF-05221304: 15 mg	Twice daily	1. Percent change from baseline in whole liver PDFF	PF-05221304 and PF-06865571 co-administration lowered steatosis by −44.6%, which was numerically greater than the reduction with PF-06865571 alone, but similar to that with PF-05221304 monotherapy	[214]
								PF-06865571: 300 mg	Twice daily			
								PF-05221304: 15 mg + PF-06865571: 300 mg	Twice daily			
								PBO	Twice daily			
NCT03938246 (FASCINATE-1)	Phase II	TVB-2640 (FASN Inhibitor)	Biopsy-proven NASH/overweight/obese/diabetic/ALT ≥ 30 U/L in patients with BMI ≤ 40 kg/m^2^ and ≥8% liver fat content on MRI-PDFF. (142)	TGs: 163 (124, 262) mg/dL Tchol: 189 (167, 225) mg/dL Apo B: 104 (89, 124) mg/dL	HbA1c: 5.8 (5.5, 6.4) % Insulin: 22 (14, 32) µU/mL HOMA-IR: 5.0 (3.7, 7.8) Glucose: 98 (80, 124) mg/dL	Hepatic inflammation	NA	TVB-2640: 25 mg	Once daily	1. Change in hepatic fat fraction from baseline in subjects with NASH by MRI PDFF 2. Safety of TVB-2640, including changes in liver enzymes by monitoring AEs	TVB-2640 treatment resulted in significant relative and absolute reductions of liver fat compared to PBO in a dose-dependent manner	[215]
								TVB-2640: 50 mg	Once daily			
								PBO	Once daily			
NCT02279524 (ARREST)	Phase II	Aramchol (SCD1 inhibitor)	Biopsy-proven NASH and BMI between 25 kg/m^2^ to 40 kg/m^2^ (247)	TGs: 1.92 ± 1.6 mmol/L Tchol: 4.88 ± 1.1 mmol/L	Glucose: 6.94 ± 2.4 mmol/L HbA1c: 6.65 ± 1.0% HOMA-IR: 9.6 ± 6.5 U	Hepatic inflammation	NA	Aramchol: 400 mg	Once daily	1. Change from baseline in mean liver fat	NASH resolution without worsening fibrosis was achieved in Aramchol 600 mg: OR = 4.74 (95% CI 0.99, 22.7)	[216]
								Aramchol: 600 mg	Once daily			
								PBO	Once daily			
NCT01265498 (FLINT)	Phase II	Obeticholic acid (FXR agonist)	Biopsy-proven NASH	TGs: 2.2 ± 1.5 mmol/l TChol 4.9 ± 1.2 mmol/l	Glucose: 6.5 ± 1.8 mmol/L Insulin: 201 ± 226 pmol/L HbA1c: 48 ± 12 mmol/mol HOMA-IR: 61 ± 74	Hepatic inflammation	NA	OCA: 25 mg	Once daily	1. Improvement in liver histology, defined as a decrease in NAFLD activity score by at least 2 points without worsening of fibrosis	OCA met primary outcome in 46% of patients compared with 21% of patients in the PBO group	[223]
								PBO	Once daily			
NCT03449446 (ATLAS)	Phase II	Cilofexor (FXR agonist) Firsocostat (ACC1/ACC2 inhibitor) Selonsertib (Authopagy inhibitor)	Advanced fibrosis (395)	TGs: 123 (101, 191) mg/dL Tchol: 177 (150, 208) mg/dL Total BAs: 7.1 (4.9, 11.6) µmol/L	Glucose: 111 (97, 138) mg/dL HbA1c: 6.0 (5.6, 6.6) % HOMA-IR: 6.2 (3.8, 8.3)	Hepatic inflammation	NA	Cilofexor:30 mg	Once daily	1. Percentage of participants experiencing treatment-emergent adverse events 2. Percentage of participants experiencing treatment-emergent laboratory abnormalities 3. Percentage of participants who achieved a ≥1-Stage improvement in fibrosis without worsening of NASH	1. Thirteen patients (3%) discontinued treatment due to an AE, with similar rates between treatment groups 2. Grade 3 and 4 laboratory abnormalities were observed in 0–13% of patients across groups 3. Differences between the treatment arms and PBO (11%) did not reach statistical significance	[225]
								Firsocostat: 20 mg	Once daily			
								Selonsertib: 18 mg	Once daily			
								Selonsertib: 18 mg + Firsocostat: 20 mg	Once daily			
								Selonsertib: 18 mg + Cilofexor:30 mg	Once daily			
								Firsocostat: 20 mg + Cilofexor:30 mg	Once daily			
								PBO	Once daily			
Antihyperglycemic drugs												
NCT01237119 (LEAN)	Phase II	Liraglutide (GLP1-RA)	Biopsy proven NASH patients with BMI ≥ 25 kg/m² (52)	Hyperlipidaemia: 9 (35%) in liraglutide group. Other parameters in liraglutide group: Esterifi ed fatty acids: 967 ± 535 μmol/L; ADIPO-IR: 22.2 ± 12.7 U; TChol: 4.5 ± 1.1 mmol/L; TGs: 1.9 ± 1.1 mmol/L	HbA1c ≤ 9% T2DM: 9 (35%) in liraglutide group; Other parameters in liraglutide group: Glucose: 6.0 ± 1.7 mmol/L; Insulin: 166 ± 80 pmol/L HOMA-IR: 6.7 ± 4.7 U	Hepatic inflammation	NA	Liraglutide: 1.8 mg	Once daily	1. Liver histological improvement	Resolution of NASH with no worsening fibrosis in 39% in liraglutide group vs. 9% in the PBO group. Progression of fibrosis 9% vs. 36%. Reduction of body weight, and BMI improvement of physical activity, and no severe AEs.	[232]
								PBO	Once daily			
NCT02970942	Phase II	Semaglutide (GLP1-RA)	Biopsy proven NASH with fibrosis stage 1, 2 or 3 patients with BMI > 25 kg/m² (320).	NA	HbA1c ≤ 10% Parameters in highest dose semaglutide group: T2DM: 49 ± 60%	Hepatic inflammation	NA	Semaglutide: 0.1 mg	Once daily	1. Percentage of participants with NASH resolution without worsening of fibrosis after 72 weeks	Percentage of patients in whom NASH resolution was achieved with no worsening of fibrosis: 40% in the 0.1-mg group, 36% in the 0.2-mg group, 59% in the 0.4-mg group (*p* < 0.001), and 17% in the PBO group. An improvement in fibrosis stage occurred in 43% of the patients in the 0.4-mg group, and in 33% of the patients in the PBO group. Mean percent weight loss was 13% in the 0.4-mg group, and 1% in the PBO group.	[233]
								Semaglutide: 0.2 mg	Once daily			
								Semaglutide: 0.4 mg	Once daily			
								PBO	Once daily			
NCT03131687	Phase II	Tirzepatide (GLP1-RA with gastric inhibitory polypeptide receptor activity)	Patients with T2DM with BMI ≥ 23 and <50 kg/m² (316)	Adiponectine: 5.1 ± 0.5 mg/L in highest dose of tirzepatide group	HbA1c: ≥7% and ≤10.5% Glucose: 164.8 ± 48.6 mg/dL in highest tirzepatide group	NA	NA	Tirzepatide: 1 mg	Once weekly	1. Change from baseline to week 26 in HbA1c Bayesian dose response.	Decreases with tizepartide were significant compared to PBO for K-18 (10 mg) and pro-C3 (15 mg) and with dulaglutide for ALT (10 and 15 mg). Adiponectine significantly increased from baseline with tizepartide compared to PBO (10 and 15 mg).	[244]
								Tirzepatide: 5 mg	Once weekly			
								Tirzepatide: 10 mg	Once weekly			
								Tirzepatide: 15 mg	Once weekly			
								Dulaglutide: 1.5 mg	Once weekly			
								PBO	Once weekly			
NCT03235050	Phase II	Cotadutide (GLP1-RA with glucagon activity) and liraglutide (GLP1-RA)	Patients with treated T2DM BMI ≥ 25 kg/m² (834)	TGs: <1000 mg/dL	HbA1c ≥ 7% and ≤10.5%	NA	NA	Cotadutide: 100 μg	Once daily	1. Change in HbA1c 2. Percent change in body weight	Cotagutide 300 μg yielded greater reduction in body weight and ALT levels vs. liraglutide. The improvement in FLI and NFS with cotadutide may indicate reduced liver fat and fibrosis, respectively.	[245]
								Cotadutide: 200 μg	Once daily			
								Cotadutide: 300 μg	Once daily			
								Liraglutide_ 1.8 mg	Once daily			
								PBO	Once daily			
NCT02686476 (E-LIFT)	NA	Empagliflozin (SGLT2i)	Patients with T2DM of age ≥ 20 years and NAFLD (100)	Parameters in the Empaglifozin group at baseline: TGs: 201 ± 124) mg/dL; HDL: 42 ± 12 mg/dL; LDL: 112 ± 35 mg/dL	HbA1c > 7.0% and <10.0% Glucose in the Emplaglifozin group at baseline: 173 ± 44 mg/dL	NA	NA	Standard care of T2DM * + Empagliflozin: 10 mg	Once daily	1. To evaluate the change in liver fat content at baseline and 3 months.	Mean MRI-PDFF difference between the empagliflozin and control groups −4.0% (*p* < 0.001).	[253]
NCT02964715	Phase IV	Empagliflozin (SGLT2i)	Obese patients with T2DM, biopsy proven NASH and BMI < 45 kg/m² (25)	Dyslipidemia 8 (88.9%); TGs: 1.6 (1.3–2.4) mmol/L; TChol: 4.4 (3.5–4.7) mmol/L	HbA1c: >6.5%	Hepatic inflammation	NA	Any anti-diabetic agent except SGLT2 inhibitors, TZDs, DPP4 inhibitors and GLP1RAs + Empagliflozin: 25 mg	Daily for 6 months	1. Change in histological grade as evaluated with non-alcoholic Steatohepatitis Clinical Research Network scoring system 2. Change in serum FGF21	Empaglifozin resulted in signifcantly greater improvements in steatosis (67% vs. 26%, *p* = 0.025), ballooning (78% vs. 34%, *p* = 0.024), and fbrosis (44% vs. 6%, *p* = 0.008) compared with PBO.	[261]
Drugs targeting immunologic system												
NCT02442687	Phase II	JKB-121 (TLR-4 antagonist)	Biopsy-proven NASH patients with BMI > 25 kg/m^2^ (65)	NA	HbA1c: ≤9%	Hepatic inflammation	NA	JKB-121: 5 mg	Twice daily	1. Reduction in liver fat content by MRI-PDFF change from baseline to week 24. 2. Reduction in liver fat content by MRI-PDFF change from baseline to week 12.	Not accomplished	[263]
								JKB-121: 10 mg	Twice daily			
								PBO	Twice daily			
NCT02316717	Phase ll	IMM-124E (anti-LPS polyclonal antibodies)	Biopsy proven NASH patients with BMI > 25 kg/m^2^ (133)	NA	HbA1c: <9%	Hepatic inflammation	NA	IMM-124E:600 mg	Three times daily	1. Incidence of AEs per arm/group. 2. Mean change from baseline in percentage fat content of the liver. measured by MRI at week 24. 3. Number of patients with treatment-related AEs. 4. Number of grade 3–5 AEs.	NA	NA
								IMM-124E:1200 mg	Three times daily			
								PBO	Three times daily			
NCT02217475 (CENTAUR study)	Phase ll	CVC (dual CCR2/CCR5 antagonist)	Biopsy-proven NASH and liver fibrosis patients with mean BMI 33.9 ± 6.5 kg/m^2^ (289)	Biomarkers in all patients at baseline TGs: 177.4 ± 130.8 mg/dLTChol: 190.2 ± 48.1 mg/dL	HbA1c: 6.54 ± 1.27%	Hepatic inflammation biomarkers at baseline in CVC group (median (min, max)): hs-CRP: 2.35 (0.2, 24.0) mg/L; IL-1β: 0.090 (0.00, 2.69) pg/mL; IL-6: 4.30 (1.4, 475.6) pg/mL; sCD14: 1731.0 (138, 3601) μg/L; sCD163: 615.0 (263, 1486) μg/L; CCL2: 499.00 (166.1, 1497.4) pg/mL; CCL4: 90.80 (2.6, 2432.9) pg/mL	NA	CVC: 150 mg	Once daily	1. Number of participant with hepatic histological improvement in NAS by ≥2 points with at least 1-point reduction in either lobular inflammation or hepatocellular ballooning, and no concurrent worsening of fibrosis at year	Improvement in fibrosis by ≥1 stage (NASH CRN system) and no worsening of steatohepatitis (no worsening of lobular inflammation or hepatocellular ballooning grade): OR = 2.201 (95% CI 1.113, 4.352)	[265]
								PBO	Once daily			
NCT03028740 (AURORA Study)	Phase III	CVC (dual CCR2/CCR5 antagonist)	Biopsy- proven NASH and stage 2 or 3 liver fibrosis patients (1779)	NA	HbA1c: ≤10%	Hepatic inflammation	NA	CVC: 150 mg	Once daily For year 1 or years 1&2	1. Superiority of CVC compared to PBO on liver histology at month 12 relative to the screening biopsy. 2. Superiority of CVC compared to PBO on the composite endpoint of histopathologic progression to cirrhosis, liver-related clinical outcomes, and all-cause mortality.	Not accomplished (study early terminated)	NA
								PBO	Once daily for year 2			
Drugs targeting microbiota												
NCT03434860	NA	Symbiter (14 strains from *Bifidobacterium*, *Lactobacillus*, *Lactococus*, *Propionibacterium* and *Acetobacter*)	T2DM patients of age 18–65 with NAFLD and BMI ≥ 25 kg/m^2^ (58)	TGs: 2.57 ± 1.03 mmol/L TChol: 6.28 ± 0.89 mmol/L	NA	Hepatic inflammation	NA	Symbiter: 10 g	Once daily	1. Changes in LFC 2. Changes in liver stiffness	Decrease of LFC, serum insulin and leptin, and IR compared with PBO	[267]
IRCT201410052394N13	Phase II	Orafti (*L. acidophilus*, *L. casei*, *L. rhamnosus*, *B. breve* and *B. longum*)	Patients of age 20–60 with NAFLD and BMI ≥ 25 kg/m^2^ (89)	NA	Glucose: 89 ± 17 mg/dL Insulin: 11.42 ± 4.5 µU/mL HOMA-IR: 2.58 ± 1.35	Hepatic inflammation	NA	Orafti: 8 g	Twice daily	1. Modulation of glycemic parameters	Decrease of LFC, serum insulin and leptin, and IR compared with PBO	[268]
NCT01791959	Phase II	Protexin (*L. acidophilus*, *L. bulgaricus*, *L. casei*, *L. rhamnosus*, *B. breve*, *B. longum and S. thermophilus*)	Patients of age > 18 with NAFLD determined by steatosis and ALT > 60 U/L (52)	NA	Glucose: 99.6 ± 24.2 mg/dL Insulin: 11.2 ± 3.4 µU/mL HOMA-IR: 2.8 ± 1	Hepatic inflammation	NA	Protexin: NA	Twice daily	1. Modulation of hepatic fibrosis, liver enzymes, and inflammatory markers	Significant decrease of serum ALT compared with PBO	[269]
IRCT201111082709N22	Phase II	Protexin (*L. acidophilus*, *L. bulgaricus*, *L. casei*, *L. rhamnosus*, *B. breve*, *B. longum and S. thermophilus*) + vitamin E	Patients of age 25–64 with NAFLD determined by steatosis and persistently elevated ALT > 30 mg/dL (60)	TGs: 162.56 ± 18.83 mg/dL TChol: 167.3 ± 18.79 mg/dL Apo B: 85.7 ± 18.2 mg/dL	Glucose: 98.63 ± 7.14 mg/dL Insulin: 1.77 ± 0.53 µU/mL HOMA-IR: 0.63 ± 0.2 µU/mL	Hepatic inflammation	NA	Protexin + vitamin E: 400 IU	Twice daily	1. Modulation of liver enzymes, leptin, lipid profile, and IR	Significantive decrease of serum ALT, leptin, plasma glucose, IR, TG, cholesterol, and LDL compared with PBO	[270]
IRCT2017020932417N2	NA	Synbiotic yogurt with *B. animalis*	Patients of age > 18 with NAFLD determined by ultrasound and grade 1–3 fatty liver (102)	TGs: 165.7 ± 60.9 mg/dL TChol: 195.3 ± 34.7 mg/dL	Glucose: 89 ± 17 mg/dL Insulin: 11.42 ± 4.5 µU/mL HOMA-IR: 2.58 ± 1.35	Hepatic inflammation	NA	NA	Three times a day	1. Hepatic steatosis and modulation of liver enzymes	Reduction of steatosis and decrease of serum ALT, AST, and GGT compared with PBO	[271]
NCT01680640 (INSYTE)	NA	Actilight with *B. animalis*	Patients with NAFLD (104)	TGs: 1.8 (1.1) mmol/L TChol: 4.9 (1.2) mmol/L	Glucose: 6.2 (2.5) mmol/L Insulin: 13.5 (7.9) µU/mL	Hepatic inflammation	Higher proportions of *Bifidobacterium* and *Faecalibacterium*, and lower proportions of *Oscillibacter* and *Allistipes* in fecal samples	NA	Twice daily	1. Modulation of liver fat content, biomarkers of liver fibrosis 2. Composition of fecal microbiome	The administration of a synbiotic altered fecal microbiome, but did not reduce liver fat content or markers of liver fibrosis compared with PBO	[272]
NA	NA	Familact (*L. acidophilus*, *L. bulgaricus*, *L. casei*, *L. rhamnosus*, *B. breve*, *B. longum* and *S. thermophilus*) + sitagliptin	Patients of age 18–60 with NAFLD determined by ultrasound and BMI 25–30 kg/m^2^ (138)	TGs: 203.84 ± 47.40 mg/dL TChol: 205.84 ± 29.29 mg/dL	Glucose: 103.25 ± 3.63 mg/dL	Hepatic inflammation	NA	Familact: 500 mg	Once daily	1. Effect of sitagliptin	Significant reduction of BMI, plasma glucose, ALT, AST, cholesterol, and TGs compared with PBO	[273]
IRCT2013122811763N15	NA	Familact (*L. acidophilus*, *L. bulgaricus*, *L. casei*, *L. rhamnosus*, *B. breve*, *B. longum* and *S. thermophilus*)	Patients of age 18–60 with NAFLD determined by ultrasound (80)	NA	NA	Hepatic inflammation	NA	Familact: 500 mg	Once daily	1. Effects of symbiotic on C-reactive protein and liver enzymes	Significantly reduced steatosis on abdominal ultrasound compared with PBO	[274]
IRCT201301223140N6	Phase II	Synbiotic (*L. acidophilus* and *B. longum*)	Patients of age 20–60 with high levels of AST and ALT and NAFLD determined by ultrasound (75)	NA	NA	NA	NA	Familact: 250 mg	Twice daily	1. Supplementation with probiotics and/or prebiotics on liver function	The treatment lowered ALT and BMI compared with PBO	[275]
NCT02496390	Phase II	FMT	Patients of age > 18 with NAFLD determined by AASLD criteria (21)	TGs: 2.30 (1.43) mmol/L TChol: 4.68 (1.15) mmol/L Apo B: 1.13 (0.35) g/L	Glucose: 7.3 (1.8) mmol/L Insulin: 196 (177) pmol/L HOMA-IR: 3.5 (1.3)	NA	No changes	FMT: 2 g	Once	1. Improvement of IR, hepatic proton density fat fraction, and intestinal permeability	Half of the patients with elevated small intestinal permeability at baseline had a signifi-cant reduction 6 weeks after allogenic transplant, coupled with an increase in GM diversity	[277]
NTR4339	NA	FMT	Patients of age 21–69 with BMI > 25 kg/m^2^, suspicion of NAFLD (elevated liver enzymes, impaired glucose tolerance and severity of steatosis on ultrasound) (21)	TGs: 1.4 ± 0.5 mmol/L TChol: 6 ± 0.8 mmol/L	Glucose: 5.8 ± 0.7 mmol/L	Hepatic inflammation	Increase in *Ruminococcus*, *Eubacterium hallii*, *Faecalibacterium*, and *Prevotella copri* in allogenic FMT. Increase in *Lachnospiraceae* in autologous FMT.	NA	NA	1. Modulation of GM composition through FMT	Allogenic FMT altered GM composition, and led to beneficial changes in plasma metabolites and genic expression involved in hepatic inflammation or lipid metabolism	[279]

Abbreviations: ACC, acetyl-coA carboxylase; AEs, adverse events; BMI, body max index; CCR, C-C motif chemokine receptor; CI, confidence interval; CRN, central research network; CVC, cenicriviroc; DGAT2, diacylglycerol O-acyltransferase 2; FASN, fatty acid synthase; FGF21, fibroblast growth factor 21; FGFR1, fibroblast growth factor receptor 1; FXR, farnesoid X receptor; GLP1-RA, flucagon-like peptide-1 receptor agonist; Hb, hemoglobin; HbA1c, glycated hemoglobin; HOMA, homeostasis model assessment; IR, insulin resistance; LFC, liver fat content; MRE, magnetic resonance elastography; MRI, magnetic resonance imaging; NA, not available; NAFLD, non-alcoholic fatty liver disease; NAS, NAFLD activity score; NASH, non-alcoholic steatohepatitis; OR, odds ratio; PDFF, proton density fat fraction; PPAR, perosyxome proliferator activated receptors; SAF-A, steatosis-activity-fibrosis activity score; SCD1, stearoyl-CoA desaturase-1; SD, standard deviation; SGLT2i, sodium glucose co-transporter 2 inhibitors; T2DM, type 2 diabetes mellitus; TChol, total cholesterol; TGs, triglycerides; TRL4, toll-like receptor 4. * Metformin, sulfonylureas, DPP-4 inhibitors, or insulin, in any combination.

## 4. Conclusions and Future Perspectives

Collectively, the hallmark of NAFLD-related pathophysiology is the hepatic ectopic fat deposition, which ultimately triggers liver injury mechanisms, and promotes or aggravates other metabolic disorders [289]. Importantly, the prevalence of this pathological event is increasing worldwide, and both lifestyle and diet play a pivotal role in its onset and progression [290]. Indeed, different European and American guidelines already recommend a reduction in body weight, weekly moderate physical activity, and hypocaloric diet as effective therapies for the treatment of this disease [18,198,291]. However, this therapeutic approach has shown some limitations, such as low adherence to the lifestyle interventions, and lack of sufficient tools to create new habits, hindering a long-term maintenance, and compromising its beneficial impact. For this reason, current efforts are focused on delving into the underlying molecular and cellular mechanism, and developing novel and promising pharmacological strategies. In this regard, some potential targets are emerging to modulate energy balance, inhibit key enzymes involved in lipogenesis, attenuate inflammation, and restore GM.

Unfortunately, despite more than a decade of extensive research focusing on NAFLD, no approved therapy for NASH currently exists. The outcomes set by clinical trials, including improvement in fibrosis and/or inflammation, are difficult to reach given the short period of time of study, drug administration, and the nature of inflammation in NAFLD, which may be chronic-relapsing or intermittent. Trial recruitment and the primary endpoints are currently based on the result of a liver biopsy, not exempt from sampling errors and intra- and inter-observer variability, so there is a need for developing non-invasive, objective, and quantitative biomarkers for the diagnosis and assessment of treatment response [292]. Furthermore, patients in the placebo arm had a striking histological response in published NASH clinical trials, probably due to the Hawthorne effect, and strict adherence to lifestyle modifications, resulting from closer patient follow-up within trials. Therefore, the placebo response should be considered, as it can confuse the results and interfere with the calculation of sample sizes and the definition of treatment endpoints.

The complexity and heterogeneity of NAFLD also represents an important impediment to the discovery of highly effective drug treatments. Clinical trials are not controlled for individual genetic predisposition, signal transduction, or metabolic profiles. Apparently, patients with predominant immunological mechanisms would benefit the most from immune system-targeting therapies. Indeed, lean patients with NAFLD also show an inflammatory profile in comparison with healthy subjects, and should not be discarded at first for immune system-targeting therapies [293]. Likewise, patients suffering from immune-mediated inflammatory diseases, such as psoriasis or inflammatory bowel disease, have shown an increased prevalence of NAFLD [294,295]. In these patients, there is a dysregulation of the immune response, with a continuous inflammatory state which might be common with NAFLD immunopathogenesis. On the other hand, NAFLD patients with T2DM or obesity seem to be the ideal candidates for antihyperglycemic drugs and lipogenesis inhibitors, respectively. However, both NAFLD and metabolic comorbidities are also well-known inflammatory conditions, so the combination of drugs with activity on different therapeutic targets could improve the outcomes [87,296]. Therefore, identifying the type of patient eligible for each type of treatment could help to unravel new pathological mechanisms and therapeutic implications. In line with this, a multi-omics data integration approach of NAFLD patients could help us to properly sub-phenotype and stratify patients, paving the way for precision medicine in NAFLD [297].

In summary, recent evidence has highlighted the role of the immunological response in NASH pathogenesis; however, there are very few clinical trials addressing the immunological components as pharmacological targets for these patients. Moreover, NAFLD is a multifactorial disorder; hence, combinatorial approaches targeting different mechanisms may enable the synergism of beneficial effects while minimizing risks. Thus, some authors have already proposed to further explore the therapeutic impact of combining anti-fibrotic drugs with compounds proven to resolve NASH, suggesting that it could result in an augmented response rate [296]. Therefore, taking into consideration the increased evidence of immunological mechanisms in NAFLD, the addition of anti-inflammatory drugs could boost the effects of this combined treatment strategy.

In addition, the effectiveness of the current therapeutic approaches could be compromised by the duration of the treatment, the disease stage of the study cohort, and the insufficiency and heterogeneity of clinical trials. Considering the last aspect, several differences in the features that define the placebo groups of the described clinical trials have been detected. Therefore, this may lead to response rate variability in this group, ultimately conditioning the final net effect observed in the therapeutic arm. In line with this, only lanifibranor OCA and pioglitazone have exhibited beneficial effects in fibrosis improvement compared to placebo, whereas semaglutide, liraglutide, and pioglitazone achieved NASH resolution in comparison to the placebo group, as described in a recent meta-analysis [296]. Accordingly, further research is required to define these critical aspects.

The evidence provided in this review could have important implications for decisions in clinical practice, highlighting the pressing need of developing effective and tolerated treatments for patients with NAFLD that also ameliorate the risk of comorbidities and complications related to this disorder.

## Data Availability

Not applicable.
